# Mirror-induced origination process of human self-consciousness—restructuring consciousness-based cognitive theory

**DOI:** 10.3389/fpsyg.2026.1679440

**Published:** 2026-06-24

**Authors:** Xiaodong Luo

**Affiliations:** 1 The Open University of Sichuan, Chengdu, China; 2 Sichuan Vocational College of Cultural Industries, Chengdu, China

**Keywords:** behavior, brain, cognition, consciousness, evolution, illusion, mirror induction, self

## Abstract

Where did “we” come from? How did our self-consciousness come to be? This great mystery has puzzled scientists for centuries. Applying representative cognitive behavioral experiments, this study proposes the Mirror-Induced Origination Hypothesis and further conducts a thought experiment to demonstrate that the brain’s conscious reaction was induced in humans’ animal ancestor to undergo illusory transformation and continuous, leap-like changes under repetitive stimulation of the ancestor’s mirror image, before transforming into mental self-consciousness (MSC). A formalized eight-stage model of conscious change—the Mirror-Induced Leap of Consciousness (MLC)—is constructed to offer a natural evolutionary account for the origin of self-consciousness and the emergence of intelligent life. The study defines consciousness as the brain’s reaction to the attribute of a percept, and elucidates that the evolutionary mechanism of self-consciousness lies in the interaction between the brain’s subjectivity and the attributes of percepts, which drives changes in conscious level and state—a mechanism that can be expressed in the form of the Consciousness Cognitive Relational Function: *C = R(a(p))*. The principle of *percept drift* is proposed to explain how repetitive mirror stimulation triggers this mechanism, and this principle is empirically supported by the rubber hand illusion (RHI) experiment. Furthermore, this study clarifies the brain’s fundamental cognitive processes underlying individual human behavior—namely sensation, perception, percept, and consciousness—and their interrelating mechanisms, offering a new paradigm for addressing the “hard problem” of consciousness in its generation, transformation, and formal definition. A comprehensive cognitive theoretical framework centered on consciousness is constructed from an evolutionary perspective, integrating key hypotheses, experimental reconstruction, logical reasoning, empirical evidence, core mechanisms and principles, modeling, and mathematization, thereby facilitating a unified understanding of cognitive science across behavioral, cognitive, and neural levels.

## Highlights

- Percept is the Processing Object Unit (POU) that the brain identifies in complex sensations.
- Consciousness gives meaning to a percept by judging its attribute.
- Self-consciousness is the brain’s reaction to its own independent subjectivity.
- Percept drift is the core illusion mechanism triggered by synchronous multisensory stimulation.
- Conscious evolution might be induced by the illusion effect of percept drift.
- The mechanism of conscious evolution is a functional relationship between the brain’s subjectivity and the attribute of a percept.
- The essence of conscious evolution is the progressive liberation of the brain’s subjectivity from the body’s instinctivity.

## 1 Introduction

A great mystery remains in the wake of evolutionism: How did our consciousness of self, making us feel so singular from other animals, emerge? Where did “we” come from? The philosopher Popper described this as the greatest miracle, an unsolved mystery, like the origin of life (Popper, 1982). A fundamental question of human existence, this issue has transcended traditional philosophy, biology, and psychology to the forefront of neuroscience (Gallagher et al., 2000) and even artificial intelligence (AI) and robotics (Chella et al., 2008). Self-consciousness was predominantly philosophers’ interest, focusing on the “self”—self-knowledge, such as Socrates’s “Know yourself” and Descartes’s mind–body dualism. As science advanced, inquiries into the nature of human beings shifted to biology, especially regarding physical origins and evolution. “Consciousness” became the subject of psychology (Baars, 2009). However, consciousness is highly subjective, and traditional attempts to examine it through introspective analysis (Seager, 2009) are “like seizing a spinning top to catch its motion” (James, 1952, p. 158). Thus, disciplines such as psychology have abandoned abstract theoretical consciousness research and turned to behaviorism (Watson, 1914), psychophysics (Green and Swets, 1966), and other fields for new empirical directions. Here, the topic of consciousness had generally been avoided as a target of scientific scrutiny (Cohen and Schooler, 1997). Recent developments in neuroscience and cognitive science (Cohen Priva and Austerweil, 2015) have begun to change this. For example, micro-level observational methods, such as brain imaging, can observe the brain without injury (Buckner et al., 1996). Computer, gene, information, big data, and virtual reality (VR) technologies (Slater et al., 2010) allow analysis of correlations among the nervous system, brain, human consciousness, and behavior.

However, the “hard problem” of how experiential self-consciousness and more primitive forms of consciousness “arise from physical systems” remains unsolved (Chalmers, 1996, p. ix, 5, 107). We still cannot definitively nor uniformly define our consciousness (Chalmers, 1996). Numerous theories discuss self-consciousness. At the macro level, self-consciousness is thought to originate from evolution (Sedekides and Skowronski, 1997), society (Wilson and Sober, 1994), and labor (Engels, 1995), among others. At the individual level, bodily self-consciousness (BSC; Blanke, 2012) connects the self and self-consciousness. At the brain cognitive level, language and symbolic cognition may link to self-consciousness emergence (Tattersall, 2004). At the neural mechanism level, the neural correlation between self-consciousness and specific brain area activation has been analyzed (Lou et al., 2017). Diverse consciousness theories adopt varied explanatory mechanisms for different targets (Signorelli et al., 2021), such as the Global Neuronal Workspace (GNW, Dehaene and Naccache, 2001), Higher-Order Thought theory (HOT, Rosenthal, 2002), and Integrated Information Theory (IIT, Oizumi et al., 2014). However, no existing model is universally accepted, either theoretically or empirically (Signorelli et al., 2021). Only a few researchers have looked beyond physicalism for philosophical and religious explanations, attributing self-consciousness origins to “a supernatural spiritual creation” (Eccles, 1989, p. xii, 249). On the other hand, “machine consciousness” (Dehaene et al., 2017) has emerged as a new focal point of debate at the inorganic level beyond humans and living beings.

As the “last frontier of science,” described by Chalmers (personal communication, April 2004), the multilevel complex representation of this Great Sphinx puzzle is prone to sink studies into the vast sea of phenomena (Gallagher, 2007) and limits them to the experiential level (Cohen and Schooler, 1997). The different paradigms of disciplines also restrict mutual understanding and inspiration between consciousness theories. In light of this, instead of addressing the traditional complex question—“What is it like” (Signorelli et al., 2021)—this study shifts its focus to the most fundamental question: How does it come into being? Starting from the basic cognitive processes of sensation, perception, percept, and consciousness at the brain cognitive level and their interrelationships, the study incorporates empirical evidence such as the mirror test (Gallup, 1970), proposes a “mirror-induced origination” hypothesis of self-consciousness, and conducts a thought experiment for reproduction and verification. In the verification process, a functionally formalized model is constructed to account for the generation and transformation of consciousness, and the emergence of self-consciousness, as well as the mechanism of conscious evolution. The rubber hand illusion (RHI) experiment (Botvinick and Cohen, 1998) is introduced to support the key links within the model.

## 2 Theoretical framework, hypotheses and empirical support

### 2.1 Redefining perception and percept

Given the lack of a unified understanding and definition of consciousness, this study focuses on the cognitive dimension of consciousness, namely the brain cognitive level. Even at this level, defining consciousness is problematic, as related ideas—including sensation, perception, percept, and attention—overlap and vaguely connect where “the boundary of sensation blurs into that of perception” (Zimbardo et al., 2014, p. 89) and “attention and consciousness form two partially overlapping sets” (Sternberg and Sternberg, 2012, p. 138). If self-consciousness is regarded as the dome of the edifice of cognitive science, then consciousness serves as the keystone of the arch that crowns the gateway, while the essential cognitive ideas—perception and sensation—constitute the arch itself and its cornerstone. Without a solid cornerstone, the arch becomes unstable, and if the keystone is incompatible with the arch, the gateway cannot be opened; such an edifice would naturally become loose—and yet this is precisely the current situation. Among these concepts, sensation—the process by which the sensory receptors and nervous system receive and represent stimulus energies from the environment (Goldstein and Cacciamani, 2022)—constitutes the most fundamental process and remains the sole one with consensus across theoretical definition, empirical validation, and neural mechanism. Therefore, these crucial ideas in the brain’s cognitive processes need to be discussed and redefined on the basis of sensation—restructuring the edifice of cognitive theory step by step, starting from a solid cornerstone.

Vagueness and divergence begin with perception and percept; the former is generally defined as the set of processes by which we recognize, organize, and make sense of the sensations we receive from environmental stimuli (Goodale, 2000). Perception is thought to bring meaning to sensation. However, for the purposes of this study, the single sensation has no meaning and does not need to be given meaning but rather the combination of specific sensations (which goes beyond the definition of sensation itself). In this study, the brain must first identify a combination of specific sensations—referred to herein as the brain’s Processing Object Unit (POU) for sensations—from the mixed and complex sensations derived from stimuli before making sense of them. It is this very sequence that constitutes the essence of perception.

How does the brain identify the POU? A typical explanation is provided by the Gestalt principles of form perception: figure-ground, proximity, similarity, continuity, closure, and symmetry based on Prägnanz’s law (Köhler, 1940). It outlines how the brain perceives objects and converts them from parts into wholes and categories into groups (Palmer, 2000). Perceptual psychologists believe the brain has specialized cells—feature detectors—to extract very specific stimuli features—an object’s length, slant, color, boundary, location, and movement (Kandel and Squire, 2000). Important empirical evidence here is the formation of visual illusions such as animal crypsis illusion—some animals can blend into their environment to go undetected by predators, for example, a lichen-mimicking katydid using camouflage to avoid being recognized and hunted by predators (Martinez-Conde and Macknik, 2016). On one hand, the katydid deceives the predator’s brain by skillfully applying Gestalt principles (figure-ground, similarity, and continuity) to form environment-like crypsis, thereby evading detection. On the other, for the predator to correctly identify a cryptic katydid, it must also utilize Gestalt principles, extracting the katydid’s shape and color from the background through symmetry. Thus, katydid and predator compete to apply Gestalt principles to influence the predator’s brain in forming POUs for sensations. This competitive application of Gestalt principles leads to the potential formation of different POUs from the same environmental sensations—the fundamental cause of illusion generation. It also indicates that the brain assigns a meaning (a katydid or a part of the environment) to the sensations only after identifying the POU. Otherwise, if the brain could directly make sense of the sensations without first identifying POUs—or if perception could directly endow sensations with meaning—it would be impossible to assign two distinct meanings to the same environmental sensations (i.e., an illusion). As reflected, the brain’s process of identifying the POU is both fundamental and important as well as separate and essential, affecting—even changing—the ultimate meaning of sensations from stimuli.

Therefore, in the brain’s complex psychological and neural processes, perception as a fundamental and separate process only results in the formation of the POU for sensations or the formation of the combination of specific, meaningless sensations; that is, the formation of percept. Thus, within the framework of the present study, perception is redefined as the process by which the brain recognizes and organizes sensations; that is, the process by which the brain recognizes and forms the combination of specific sensations from sensations received from environmental stimuli. Accordingly, the percept is defined as the product of the brain’s recognition and organization of sensations; namely, the combination of specific sensations recognized and formed by the brain from sensations received from environmental stimuli—the brain’s Processing Object Unit (POU) for sensations.

In actual cognitive experience, affected by the complexity of environmental stimuli, the brain tends to form multiple POUs simultaneously, and the core processing mechanism adopted by the brain for this scenario is selective processing, which constitutes attention. From the theoretical perspective of the present study, attention is defined as the brain’s selection of percept; this definition is consistent with the standard theoretical interpretation of selective information processing in the field of cognitive science (Eysenck and Keane, 2015; Pashler, 1998). Furthermore, the meaning (i.e., interpretation) of percept should be given by consciousness, a higher-level psychological and neural process in the brain. Otherwise, if perception is meaningful, consciousness is meaningless, which is why consciousness is difficult to define. Conversely, consciousness is meaningful only when perception is meaningless.

### 2.2 Definition and formula of consciousness

How should consciousness be understood and defined? At the neural mechanisms level, the GNW (Dehaene and Naccache, 2001) theory regards consciousness as information that is coordinately activated and widely propagated by distributed neuronal populations in the brain’s global workspace. By contrast, the HOT (Rosenthal, 2002) theory, at the brain cognitive level, takes consciousness as the representation of lower-order mental states by higher-order thoughts. The IIT (Oizumi et al., 2014), in turn, treats consciousness as an irreducible set of concepts at the level of individual experience. This study focuses on the following question: How is consciousness generated at the brain cognitive level? (Applying the principle of a “generative definition” of formal logic and math, if a fundamental concept is difficult to define in terms of existence, it can be defined by describing its unique formation process; see Qiu, 2002). Consider the most elementary scenario of consciousness-generating: How does consciousness arise when the brain is confronted with an unfamiliar object in an unfamiliar environment? Following a general understanding of conscious experience and the aforementioned definition of percept, consciousness generation at the cognitive level occurs when the brain first perceives the complex sensations in the environment and forms a certain percept associated with an object (i.e., identifies specific sensations associated with the object from complex environment sensations, forming a certain POU), subsequently making an attribute judgment of what the certain percept “is” and “can do,” and the state judgment and anticipation of what it “did,” “is doing,” and “will do,” for the brain to decide what the brain “should do.” Applying inductive reasoning, consciousness is defined as the brain’s reaction to the attribute of percept, including the state of this attribute. More abstractly, if percept is treated as a function of space, attribute as a function of time, and attribute state as a value of function, then consciousness is a spatial–temporal response of the brain (i.e., nerves). Simultaneously, the process by which the brain generates consciousness is the conscious reaction process (CRP).

According to this definition, the “brain,” the “attribute of percept” (including the attribute’s state), and the “consciousness” generated by the brain’s reaction are the subject, object, and outcome of consciousness, respectively—the three elements of consciousness. Thus, the CRP can be mathematically expressed as:


Brain→Attribute of percept=Consciousness (1)

Equation 1 is named the Conscious Reaction Formula (CRF)—the basic formula for the process of consciousness generation (i.e., the brain’s conscious reaction)—where “**→**” means “(brain)‘s reaction to”. As will be shown in the subsequent analyses and applications, all components in this formula are variables. These variables’ different states can be used as different values to describe distinct elements and states of consciousness. The changes in these variables also reflect changes in consciousness elements, followed by changes in consciousness. Thus, the CRF can be applied to explain different states of, levels of, and complex changes in consciousness.

### 2.3 Hypotheses and empirical support

The new definitions of consciousness and related concepts involve integration, simplification, focus of the original definitions’ content per existing cognitive science theories, and restructuring of mutual logical relations. Neither conflict with various phenomena and experiments associated with the original definitions and provide newer, more elaborate explanations. Experimental and phenomenological research results also support new theoretical definitions of these basic concepts.

Recently, at the neural mechanism level, consciousness and related cognitive processes have been extensively investigated with advanced cognitive neuroscience methods (Dehaene and Changeux, 2011). For example, neuronal mechanism research has found that information from different senses is integrated to improve perception (Fetsch et al., 2013). The brain’s bimodal and multimodal neurons have important neurophysiological features of multisensory bodily perception: They respond to stimuli via multimodality (e.g., tactile, visual, auditory, and proprioceptive cues; Andersen, 1997). In this study, multisensory integration at the neural mechanism level represents the brain’s recognition and organization of sensations when multisensory integration is projected and reflected on the brain cognitive level—consistent with the definitions of perception and percept proposed above. Hence, bimodal and multimodal neurons, multisensory integration, and feature detectors that discern the unique features of a single sensory stimulus can empirically support the percept and perception definitions at the neural mechanism level (Deroy et al., 2016). Further, they provide the basis for understanding the neural mechanisms of more complex cognitive processes such as consciousness and attention (Spence and Driver, 2004).

At the individual behavior level, the mirror test is a typical behavioral experiment judging whether an animal recognizes its mirror image and itself (see the Glossary for details). These tests have shown most animals (including monkeys, e.g., rhesus macaques) cannot identify themselves in a mirror, considering the mirror image as a conspecific (Anderson, 1994; Anderson and Gallup, 2015); these creatures are majority-failed-test (MFT) animals. Only humans and a few animals [e.g., chimpanzees (Gallup, 1970), elephants (Plotnik et al., 2006), dolphins (Reiss and Marino, 2001)] can pass the mirror test; these creatures are handful-passed-test (HPT) animals. This behavior is the reaction of the animal’s brain to its mirror image, referred to as the mirror reaction. In sum, humans’ mirror reactions differ from that of MFT animals. HPT animals are discussed in the conclusions.

How does the mirror test relate to self-consciousness and consciousness? Can the mirror test be explained theoretically? To answer these, this study proposes theoretical hypotheses, definitions, and inferences.

*Hypothesis 1*: The mirror reaction is a manifestation of consciousness.

As the definition of the mirror reaction aligns with consciousness in this study, this hypothesis holds under the study conditions. Further, even under the traditional understanding of consciousness, this hypothesis is reasonable and largely accepted. Hence, it establishes the consistency of the theoretical definition of consciousness with typical behavioral experimental performance.

*Hypothesis 2*: Different mirror reactions are attributable to different levels of consciousness between humans and MFT animals.

How different are levels of consciousness between humans and MFT animals? This can be determined by excluding the similarities of both. Humans and MFT animals can perceive their own bodies: The brain can recognize and organize the body’s sensations, including vestibular and kinesthetic senses (Zimbardo et al., 2014), to form the percept of the body, named the bodily self (consistent with “bodily self” in neuropsychology). Typical behaviors based on this include accurately perceiving and controlling one’s body and moving purposefully (Legrand, 2006). Evidently, this reaction is brain-based and fits the definition of consciousness; therefore, it is a conscious reaction, named herein as BSC, consistent with the cognitive neuroscience term (Tsakiris et al., 2007). Based on the definition of consciousness, BSC is defined as the brain’s reaction to the attribute of the body it inhabits, including the state of this attribute. Thus,


Brain→Attribute of bodily self=BSC (2)

This definition is compatible with cognitive neuroscience’s understanding—BSC includes body-centered perception (hand, face, trunk) based on multisensory brain mechanisms underlying the integration of bodily signals (proprioceptive, vestibular, visual bodily inputs; Blanke et al., 2015). The brain mechanisms and particular neurons for multisensory integration have long been known in neurophysiological research on non-human primates (Rizzolatti et al., 1981). Human neuroimaging studies have demonstrated consistency between activity in major brain areas of BSC (e.g., hand-related multisensory integration in the premotor cortex) and neurophysiological data from non-human primates in these areas, followed by reviewed human behavioral data (Lopez and Blanke, 2011). Thus, supported by the micro level of neurons and neural mechanism, meso level of brain region functions and cognitive, and macro level of individual behaviors and the mirror test, humans and MFT animals possess equivalent BSC. This is precisely the shared level of consciousness—the similarity—between humans and MFT animals. Hence, if this is excluded, the gap between the levels of humans’ and MFT animals’ consciousness can be determined.

The difference is obvious: A direct mirror test result is that humans can recognize their own mirror images while MFT animals cannot. Introducing BSC’s definition, such a gap between humans and MFT animals is reflected: Although MFT animals cannot recognize themselves or mirror images of themselves, they possess BSC, can recognize their bodily selves, and have a consistent brain mechanism linked to BSC, as is in humans. Humans can recognize themselves, mirror images of themselves, and their bodily selves. Hence, the traditional “*self*” is not equal to *bodily self* but apparently includes *bodily self*. That is,


Self≠Bodily self (3)

Self⊃Bodily self (4)

Thus, the gap is that the *self* has an additional special connotation compared to the *bodily self*, which is vital for identifying and recognizing the *self*, referred to as the *mental self*. Therefore,


Mental self=Self–Bodily self (5)

Self=Bodily self+Mental self (6)

Excluding the same level of BSC for humans and MFT animals, the essential meaning of the mirror test can be revealed, leading to

*Hypothesis 3*: The gap between the consciousness levels of humans and MFT animals is that humans can recognize their mental self and thus possess the consciousness of a mental self [named mental self-consciousness (MSC)], whereas MFT animals cannot.

Thus,


Brain→Attribute of mental self=MSC (7)

Empirical evidence derived from primatological research, particularly comparative genomic studies, demonstrates that humans and non-human primate MFT animals share consistent taxonomic characteristics within the order Primates and exhibit a high degree of genomic similarity. For example, humans share approximately 93% of their DNA with rhesus macaques (Rhesus Macaque Genome Sequencing and Analysis Consortium, 2007). This presents a striking contrast to the pronounced cognitive disparities—including divergent mirror reactions (Anderson and Gallup, 2015)—between humans and MFT animals, and thus indirectly lends support to Hypothesis 3 from a reverse perspective: Humans most likely possess a unique trait absent in MFT animals, which, from the perspective of this study, is MSC.

This raises the following question: How did humans acquire their MSC? According to the observed macroevolutionary trend from simpler to more complex forms, it is reasonable that modern, self-conscious humans also evolved from their ancient, non-self-conscious animal ancestor (Dobzhansky, 1967). Obviously, *humans’ animal ancestor* referred to in this study is reasonably categorized as one of the MFT animals: as empirical evidence, humans and rhesus macaques shared their last common ancestor approximately 25 million years ago (Kumar and Hedges, 1998); logically, the performance of this common ancestor on the mirror test could not have surpassed that of modern rhesus macaques, meaning it was also one of MFT animals, just as macaques are today. The question therefore becomes: When and how did *the human ancestor* first acquire MSC? Again, the mirror test and hypotheses 1–3 can be applied: The human ancestor who acquired MSC could pass the mirror test, whereas the earlier animal ancestor of humans—as we may reasonably assume, originated from the initial divergence of the common ancestor of humans and rhesus macaques approximately 25 million years ago—could not. Thus, the question of how humans’ animal ancestor evolved into the human ancestor who first acquired MSC can be simplified to how humans’ animal ancestor crossed the threshold of “self-recognition” when confronted with the mirror test. Further, this study rather daringly speculates that

*Hypothesis 4*: It was precisely when confronted with its own mirror image that humans’ animal ancestor was induced to eventually accomplish the crucial leap of “self‑recognition.” — the Mirror‑Induced Origination Hypothesis of human MSC.

The extended mirror test provides experimental support for the theoretical Mirror-Induced Origination Hypothesis. In Chang et al. (2015), rhesus monkeys passed the mirror test after repeated training in a mirror environment (see the Glossary for details). This does not deny monkeys are incapable of mirror self-recognition but rather provides the possibility of a change from conditioned responses to mirror-induced self-directed behaviors—from being incapable of self-recognition to acquiring self-recognition. The monkeys’ self-face-recognition after mirror training resembles the self-recognition acquired by humans’ animal ancestor through “leaping,” as described above. Its relationship with the recognition of the mental self and the possession of MSC in Hypothesis 3 remains to be clarified. This shows that the ability and neural mechanism of self-recognition are not entirely determined by congenital species factors but can be changed and acquired, and the mirror environment is an important condition (Suddendorf and Butler, 2013), especially for vision-dominant animals. It provides an experimental possibility for humans’ animal ancestor to leap from non-self-recognition to self-recognition in the mirror environment, the key for the tenability of the Mirror-Induced Origination Hypothesis. This is significant in higher animal evolution research, especially the evolution of consciousness.

The effectiveness of synchronous multisensory stimulation in this experiment reinforces the mutual proof between theory and the neural mechanism of consciousness multisensory integration (Chang et al., 2015). Long-term synchronous multisensory stimulation can alter bodily signals’ multisensory integration and may lead to changes in body ownership, self-identification, and self-location (e.g., body illusions, body-part illusions); this is linked to the plasticity mechanism of bodily signals’ multisensory integration (Blanke et al., 2015). The synchronous visual-tactile stimulation in this monkey-training may have activated the face-selective bimodal neurons in the ventral intraparietal brain area (Ishida et al., 2010); systemic and long-term activation might have led to changes in neurons and established a new systemic link between the visual (i.e., the mirror) and tactile face (i.e., the self), resulting in successful self-face recognition (Blanke et al., 2015).

In sum, the theoretical possibility of the leap of self-recognition in the Mirror-Induced Origination Hypothesis is supported by both experimental and neural mechanisms; that is, the Mirror-Induced Origination Hypothesis of human MSC is both theoretically and experimentally possible.

## 3 Experimental reproduction

The next question is: Can this theoretical hypothesis be experimentally verified? As it is difficult to reproduce the process of humans’ animal ancestor acquiring self-recognition and then MSC in evolutionary history, a thought experiment is used.

### 3.1 Lakeside Image thought experiment

Per the Mirror-Induced Origination Hypothesis, an imagined animal ancestor of humans is the experimental participant and placed in a mirror environment as the experimental condition. Changes are “detected” in the participant’s individual behavior and brain’s conscious reaction using deductive reasoning, checking whether MSC was generated. The brain’s conscious reaction and related elements are taken as the experimental variables and detection indicators, focusing on the changes they undergo as the experimental data.

**Figure 1:**
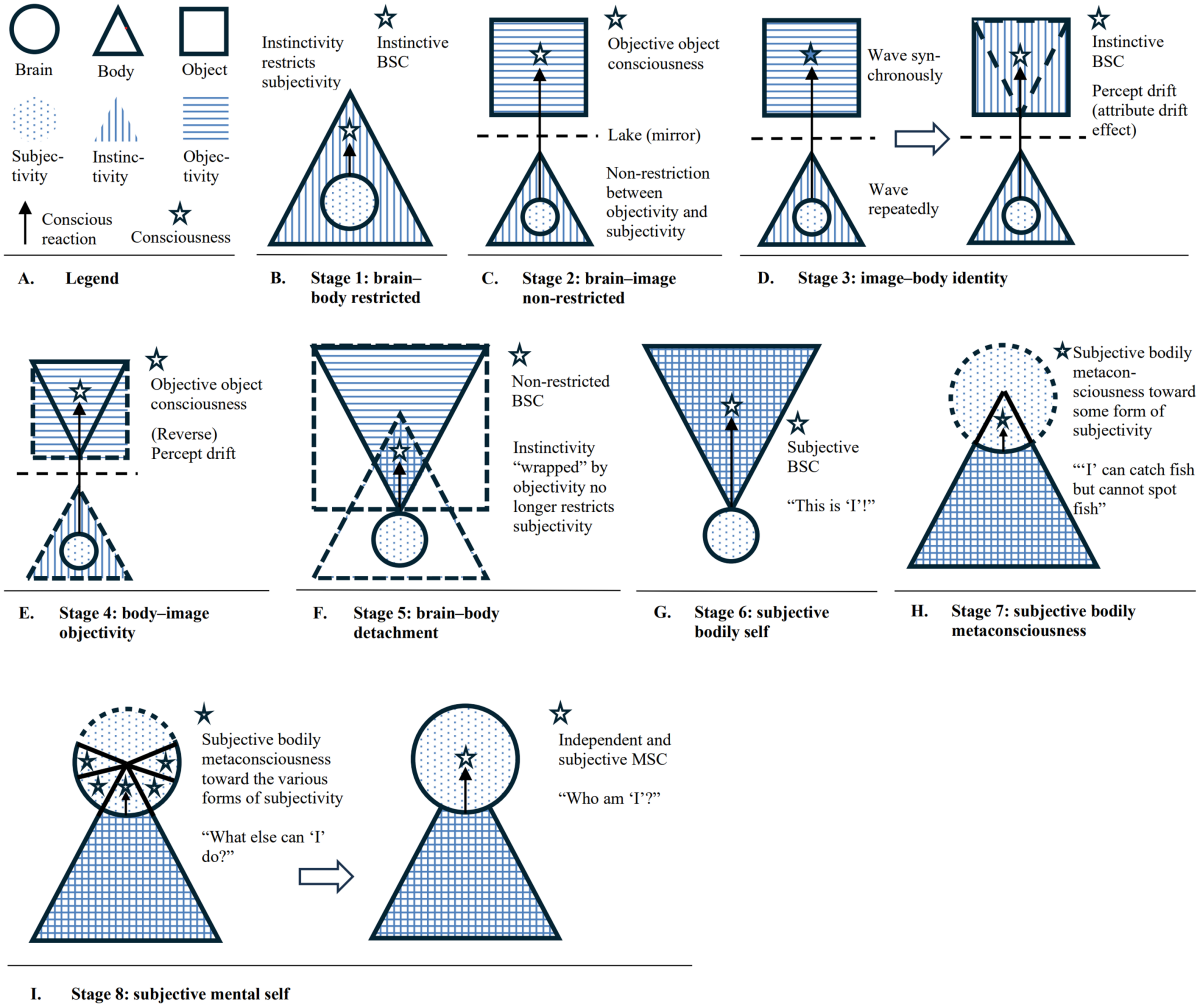
Lakeside Image thought experiment: eight‑stage process (labeled B-I; see Section 4.2) and analysis of self-consciousness’ mirror-induced origination.

This thought experiment is named “Lakeside Image,” and its process is as follows^1^ (see Figure 1 for visualization presentation): Imagine that 1 day in evolutionary history, an animal ancestor of humans (Figures 1A,B) saw an image of itself in a lake (Figure 1C). It would attempt to get rid of the image by waving its hand repeatedly as if the image was a conspecific, only to find of course that the image kept imitating its movements (Figure 1D left). In an illusion-like state of confusion, the animal probably thought it was its own intention to move its hand and that of the image synchronously. Thus, it focused on the image’s hand and slowly, tentatively controlled the image, intending for it to repeat its movements. Obviously, what the animal wanted to do (such as drinking water with its hand), the image did, achieving what it wanted just as freely as its own body normally did. Suddenly, the animal realized that this image was not another conspecific, but the body it controls (Figure 1D right). The body was this image (Figure 1E), a completely independent individual (Figure 1F), and a special individual who could be freely controlled by itself. “Ah!” s/he^2^ was likely tempted to exclaim, “This is ‘I’!”^3^ s/he—whom I name David—would think (Figure 1G). Thus, “I” was born, and self-recognition came to be. Then, another conspecific—whom I name Ruby—came to the lakeside. Looking at Ruby and her gaze, David thought, “Ruby has spotted a fish. Where is the fish?” Following the direction of Ruby’s gaze, David found a large fish and caught it. Looking at his own shadow and Ruby’s shadow in the water, David thought, “This is ‘I’, and that is Ruby. ‘I’ can catch fish, but Ruby cannot; Ruby can spot fish, but ‘I’ cannot. We can catch more fish together”. David then shared the fish with Ruby and they formed a partnership (Figure 1H). In this way, David cultivated more partners. One day, they all came to the lakeside. Gazing at the reflections of his partners and himself in the water, David mused, “Jimmy can do this but not that; Kitty can do that but not this; Ruby can do those things but not these; ‘I’ cannot do those things but can do these… What else can ‘I’ do?” (Figure 1I left) In an instant, David posed the fundamental question, “Who am ‘I’?” (Figure 1I right). Accordingly, MSC emerged.

### 3.2 Experimental interpretation

A key step in this thought experiment is that the animal ancestor of humans waves repetitively at its lakeside mirror image, similar to synchronous stimulation in the RHI experiment and producing a similar illusion effect (see Section 4.2.3. for details). S/he thus identifies the mirror image as her/his own body (BSC is transformed from the first-person to the third-person perspective), generating a judgment of “this is ‘I’” in the brain, indicating the emergence of self-recognition. In the subsequent stage, the brain forms the cognition of “can catch fish” with regard to the “I,” indicating the emergence of metacognition (Flavell, 1979; Smith et al., 2009). The final outcome is that the brain poses the question “Who am ‘I’?”, symbolizing the genesis of self-consciousness (MSC). The results of this experiment demonstrate that human MSC arose in a mirror environment. Moreover, the experimental process leading to this shows the specific process of the origination of human MSC in a mirror environment—a complex process wherein the brain’s conscious reaction, induced by repetitive mirror stimulation, undergoes illusory transformation and continuous progressive changes, culminating in MSC (see Section 4.2. for detailed argumentation). This origination process is termed the Mirror-Induced Leap of Consciousness (MLC). The emergence of MSC represents a logically inevitable outcome deduced from changes in the brain’s conscious reaction, as inferred from the participant’s behavioral changes and brain cognitive changes under the experimental conditions.

## 4 Model construction and justification

How should these experimental results be verified? Can the MLC process underlying the origination of human MSC be fully explained and theoretically justified? The following section details a multi-stage consciousness change model aiming to describe, analyze, derive, justify, and explain the entire MLC process, including experimental support and verification for its key stages.

### 4.1 Model framework

First, the mathematization of experimental phenomena is conducted by abstracting and transforming them at the macro level of individual behavior in the “Lakeside Image” into states of both consciousness and elements (i.e., the subject, object, and outcome of consciousness) at the brain cognitive level. Subsequently, the CRF is applied to mathematically model the states of the three elements of consciousness and actual states of consciousness as numerical values of experimental variables and their interrelationships. This approach allows the various phenomena and states in the experiment to be mathematically described.

Second, the mathematical decomposition of the entire experiment is examined. CRFs are applied to the states of consciousness throughout the entire experiment process of the participant; this enables the entire experimental process (including the final result) and the demonstrated MLC process to be expressed as continuous changes in the brain’s conscious reactions. Here, the complex MLC process can be mathematically decomposed into eight simple and ordered single CRPs, abstractly expressed by CRFs. This decomposition is based on the changing process of the conscious reactions of the participant’s brain revealed by deductive reasoning in the “Lakeside Image.” Thus, the process and result of the “Lakeside Image” are mathematized and modeled.

Third, the deductive reasoning and functional interpretation of the experimental change processes in states and phenomena are explored by applying the eight CRFs and their elements. Logically, the brain must recognize three kinds of basic existence (basic percept)—its body, objects outside its body (outside objects), and the brain itself (as cognitive function)—these have three different fundamental attributes: instinctivity, objectivity, and subjectivity, respectively. Under the experimental conditions (especially the repetitive mirror image stimulation), the interaction of the three elements of the experimental participant’s consciousness and the three attributes induce changes in the brain’s consciousness. This leads to CRPs transformations, resulting in MSC generation. This transformational mechanism is further interpreted in functional formulation.

Fourth, the verification of key stages is examined. How does repetitive mirror stimulation induce changes in the brain’s consciousness? This study shows the RHI as robust experimental evidence, revealing that the cognitive mechanism common to both mirror-induction and RHI is percept drift. This mechanism drives the critical illusory transformation and consciousness changes within the brain in the RHI and “Lakeside Image” thought experiment. The corresponding neural basis is also briefly discussed.

### 4.2 Detailed derivation and justification verified by the RHI experiment

Based on the changes in the participant’s behavioral manifestations, cognitive phenomena, and corresponding brain conscious states during the experiment, the MLC process is divided into eight stages.

#### 4.2.1 Stage 1: brain–body restricted

This stage precedes the “Lakeside Image” and is therefore the original state before MSC emerged. Humans’ animal ancestors, whose evolutionary level of consciousness was still animalistic, possess the same BSC as other MFT animals. As per the CRF, the object of their BSC is the attribute of their own bodies as a percept. By analyzing theories of animal behavior, the fundamental attribute of the body percept is instinctivity—the attribute of innate existence. Significantly, determined by the animal body’s biological heredity and physiological evolution, this percept: (1) is formed by the body’s instinctive impulse, perceived by the brain, such as innately perceiving the body’s position; (2) can stimulate the body’s instinctive reactions such as stimulating glandular secretion; and (3) is linked to the body’s instinctive needs, such as balance or food. The brain, the subject of consciousness, can purposefully control the body. Its fundamental attribute is subjectivity, the attribute of self-generation, which can produce a subjective purpose and impulses, such as coordinating various parts of the body to maintain balance or multiple organs for food intake. This is the source of consciousness. Crucially, in the complete animal stage, the brain’s subjectivity is restricted by the body percept’s instinctivity. Specifically, the brain’s subjective purpose is to meet the body’s instinctive needs, and its subjective impulses must be performed and realized by the body’s instinctive impulses. This is named the *restriction of instinctivity on subjectivity*,^4^ the basic feature of the brain’s conscious evolutionary level determined by the brain’s physiological evolutionary level in the animal stage.^5^ Therefore, as the outcome of consciousness, the BSC in this stage can be named *instinctive BSC*.

Hence, the CRP’s reflection of this stage’s characteristics can be expressed as: The brain, whose subjectivity is restricted by instinctivity, generates instinctive BSC toward the instinctivity of the body percept (Figure 1B). This stage is referred to as “brain–body restricted.” This CRP can be abstractly expressed as a simplified CRF:


$$\begin{array}{l} \text{Brain}\;(\text{subjectivity}\subseteq \text{instinctivity})\to \text{Instinctivity}\;(\text{body})\\ =\text{Instinctive}\;\mathrm{BSC} \end{array}$$ (8)

Where “⊆” means “is restricted by” (the same as the following).

#### 4.2.2 Stage 2: brain–image non-restricted

This stage begins the “Lakeside Image.” “One day in evolutionary history, an animal ancestor of humans saw an image of itself in a lake” (see text footnote 1, respectively). As an experimental participant, at the brain cognitive level, this animal (whose evolutionary level of consciousness corresponded to the animal stage) notices its mirror image (Figure 1C). The brain perceives the image in the same way as conspecifics, prey, trees, and other outside objects. The brain’s reaction to the outside object’s attribute, including the attribute’s state, is object consciousness. The fundamental attribute of the outside-object percept is objectivity—the attribute of objective existence. The first implication is that the objectivity of percept cannot restrict or control the brain’s subjectivity, and the second is that the brain’s subjectivity cannot control or influence the objectivity of percept directly. As per the restriction between instinctivity and subjectivity, the relationship is named *non-restriction between objectivity and subjectivity*. Hence, the brain treats the image “as if the image was a conspecific” (see text footnote 1, respectively); the outcome of consciousness is named *objective object consciousness* (Figure 1C). At the level of individual behavior, it is expressed as: “It would attempt to get rid of the image by waving its hand repeatedly” (see text footnote 1, respectively).

Thus, this stage’s CRP is expressed as: The brain, whose subjectivity is non-restricted with objectivity, generates objective object consciousness toward the objectivity of the image percept (Figure 1C). This stage is referred to as “brain–image non-restricted.” The corresponding simplified CRF is expressed as:


Brain(subjectivity⊆objectivity)→Objectivity(image)=Objective object consciousness (9)

Where “⊈” means “is non-restricted with”.

#### 4.2.3 RHI experiment and stage 3: image–body identity

An important change occurs in this stage. The animal ancestor’s image “kept imitating its movements” (see text footnote 1, respectively). The movement of the outside object (the image’s hand) is synchronized with that of the bodily self (its own hand; Figure 1D left). These significant visual and motion-related similarities are likely to cause “an illusion-like state of confusion” (see text footnote 1, respectively) in the brain, that is, whether its own (the brain’s) subjective intention caused the synchronized movements of the image’s hand while directing the movement of its own hand. Possibly, “the animal probably thought it was its own intention to move its hand and that of the image synchronously” (see text footnote 1, respectively). “Thus, it focused on the image’s hand and slowly, tentatively controlled the image, intending for it to repeat its movements” (see text footnote 1, respectively). When the brain shifts the focus of subjective intentions to the image’s hand and both the intended actions and desired effects of the subjective intention can be achieved by the image’s hand in succession, an important change occurs: The brain can “control” the image according to its own subjective intentions (the effect of synchronizing the movement of the image’s hand with that of its own hand). Here, “its (the brain’s) subjective impulse must (could) be performed and realized by the body’s (image’s) instinctive impulse (action)” (consistent with the description of the definition referenced to in text footnote 1, respectively). Moreover, “what the animal wanted to do (such as drinking water with its hand), the image did” (see text footnote 1, respectively) (a multisensory integration effect in the brain of the visual effect of “drinking water” with the image’s hand and the instinctive effect of “drinking water” with its own hand).

Enabled by the image, “the brain’s subjective purpose is (achieved) to meet the body’s instinctive needs” (consistent with the description of the definition referenced to in text footnote 4, respectively). It appears very similar to when the image “took over” the body and achieves “the restriction of instinctivity (of the body) on subjectivity (of the brain)” (see text footnote 4, respectively). Here, the brain subjectively identifies the relationship between the attribute of the image percept and the brain’s subjectivity with the restrictive relationship between the instinctivity of the body percept and the brain’s subjectivity; it thereby identifies the attribute of the image percept with that of the body percept (i.e., instinctivity) and then identifies the image percept with the body percept. This process is named *percept drift* (Figure 1D right)—the brain subjectively identifies different percepts as the same owing to the *synchronous connection* triggered by similarity between sensations from stimuli (such as vision and motion) derived from perceptual objects and the brain’s memory and experience regarding sensations forming certain percepts. This process comprises three steps: Owing to the synchronous connection, the relationship between the brain’s subjectivity and the attribute of percept B is identified with the relationship between the brain’s subjectivity and the attribute of percept A (*relationship identification*). Hence, the attribute of percept B is identified with that of percept A (*attribute identification*). Subsequently, percept B is identified with percept A (*percept identification*). This constitutes an illusory process that results in an effect wherein the brain deems percept B to possess attributes of percept A, similar to attributes of percept A drifting toward percept B. This phenomenon is thus termed the *attribute drift effect*.

The RHI is a typical experiment supporting percept drift and resulting attribute drift effect, first reported by Botvinick and Cohen (1998). They hid participants’ hand from their line of sight and placed a rubber hand at the corresponding line of sight position. The real and rubber hand were brushed synchronously for 10 min (*synchronous stimulation*; author note). Participants perceived the tactile sense from the rubber hand (*perceptual illusion*; author note), felt as if the rubber hand was part of their body (*body ownership change*), and perceived the position of their real hand shifted toward the rubber hand (*proprioceptive drift*). Similar experiments include the RHI (Tsakiris and Haggard, 2005), enfacement illusion (Tsakiris, 2008), full-body illusion (Aspell et al., 2013), out-of-body illusion (Ehrsson, 2007), and body-swap illusion (Petkova and Ehrsson, 2008).

Empirically, the RHI supports the process and effect of percept drift. First, *the synchronous stimulation confirms the relationship identification in percept drift*; the visual-tactile synchronous stimulation to two similar but different percepts of the real and fake hands in the RHI triggers the synchronous connection and subsequently establishes the identification of two different relationships between the brain’s subjectivity and the attributes of two percepts (A and B) in percept drift. Second, *the perceptual illusion confirms the attribute identification in percept drift*; the illusion that the brain perceives the tactile sense of the real hand from the visual fake hand in the RHI is essentially the attribute identification that causes the attribute of percept B (fake hand) to be identified with that (tactile sense) of percept A (real hand) in percept drift. Third, *the body ownership change confirms the percept identification in percept drift*. In the RHI, the brain perceiving the fake hand as part of the body—the body ownership of the real hand percept is changed to an illusory ownership of the fake hand percept—is the percept identification where percept B (fake hand) is identified with percept A (real hand) in percept drift. Fourth, *the proprioceptive drift and similar effects confirm the attribute drift effect of percept drift*. In the RHI, the brain perceives the attributes of the real hand percept (proprioceptive position) drift toward the fake hand percept—the effect of percept A’s attributes drifting toward percept B in percept drift.

RHI’s neural mechanisms have been extensively studied. Research has associated the RHI with the multisensory integration brain mechanisms of bodily signals underlying BSC; the illusory states of BSC can be successfully induced by artificially manipulating multisensory stimulations like vision-touch (Blanke et al., 2015). Multisensory neuron research has demonstrated that the RHI (or similar illusions) can be induced in humans and MFT non-human primates (Graziano, 1999; Graziano et al., 2000). Researchers have applied conditions similar to the multisensory stimulation used to induce the RHI in humans (i.e., synchronous visual-tactile stroking of the monkey’s hidden, real arm and a visible, realistic, fake arm) to test the response characteristics of the multimodal neurons in area 5 of the parietal lobe of the monkey brain. Prolonged and repeated visual-tactile stroking affect the neurons’ tuning characteristics; they are tuned to the false arm’s visual location, causing them to respond like in the RHI. In addition to tactile, visual, and auditory signals, multisensory neurons integrate proprioceptive (and sometimes vestibular) signals, allowing their multisensory receptive field to be anchored to a certain body part (Blanke et al., 2015). If the specific body part moves, the spatial location of the visual or auditory receptive field shifts concertedly from the original spatial location to a new one (Graziano et al., 1997). Perhaps in the RHI (or similar illusions), the visual-tactile stroking procedures for the specific body part to which the tactile receptive field is anchored change the neural responses of the corresponding multimodal neurons, leading to displacement or expansion of their visual or auditory receptive fields (Blanke et al., 2015). Hence, these multimodal neurons encode (and produce illusory responses such as proprioceptive drift toward) the seen (fake) hand, face, or whole body, even in a location beyond the boundaries of the real body (Iriki et al., 2001; Noel et al., 2015).

We can infer that humans’ animal ancestors that were close to acquiring MSC, with a level of consciousness between modern humans and MFT non-human primates, had the same brain neural mechanisms and neuronal basis that could lead to the RHI (or similar illusions of the face, body, etc.). This precisely led to the percept drift of humans’ animal ancestor in the Lakeside Image thought experiment. Here, “the image kept imitating its (own hand) movements” (see text footnote 1, respectively), triggering the synchronous connection between the visual-motional sensations of the image’s hand and the visual-motional sensations (memories) of its own hand in the brain; this served as a synchronous stimulation of the RHI, inducing an RHI-like percept drift and attribute drift effect. Further, percept drift in this study is a theoretical explanation for the RHI at the brain cognitive level based on the MSC concept system; percept drift and RHI are mutually corroborated theoretically and experimentally. Thus, owing to the percept drift and attribute drift effect, the brain suddenly realizes “this image was not another conspecific, but the body it controls” (see text footnote 1, respectively) and generates the instinctive BSC toward the attribute of the image percept (Figure 1D right).

Hence, this stage’s CRP is expressed as: The brain, whose subjectivity is restricted by instinctivity, generates instinctive BSC toward the instinctivity of the image percept that is subjectively identified with the body percept (Figure 1D right). This stage is referred to as “image–body identity.” The corresponding simplified CRF is:


$$\begin{array}{l} \text{Brain}\;(\text{subjectivity}\subseteq \text{instinctivity})\to \text{Instinctivity}\;\\ (\text{image}=\text{body})=\text{Instinctive}\;\mathrm{BSC} \end{array}$$ (10)

#### 4.2.4 Stage 4: body–image objectivity

This stage involves continuous changes. Since the image has an objective existence, it is likely that memory, instantly between thoughts, reminds the brain of the image’s objectivity. Given the synchronous connection effect of the image–body association formed in the image–body identity, percept drift is again triggered in the brain. This time, the relationship between the attribute of the identified body percept and the brain’s subjectivity is synchronously identified with the non-restrictive relationship between the image percept’s objectivity and the brain’s subjectivity (relationship identification). Further, the brain subjectively identifies the body percept’s attribute with the objectivity of the image percept (attribute identification) and then identifies the body percept with the image percept (objective object, percept identification; Figure 1E). This process can be regarded as the reverse process of percept drift in the stage of image–body identity or that percept drift is reversible. The brain realizes “the body was this image” (see text footnote 1, respectively), generating object consciousness toward the objectivity of the body percept (Figure 1E). In behavioral observations of animals, their generation of objective consciousness of a certain part of their body facilitates understanding of the body’s object consciousness. For example, a kitten chases its tail as prey or toy.

Hence, this stage’s CRP is expressed as: The brain, whose subjectivity is non-restricted with objectivity, generates objective object consciousness toward the objectivity of the body percept that is subjectively identified with the image percept (Figure 1E). This stage is referred to as “body–image objectivity.” The simplified CRF is:


Brain(subjectivity⊆objectivity)→Objectivity(body=image)=Objective object consciousness (11)

#### 4.2.5 Stage 5: brain–body detachment

In this stage, the brain generates two kinds of conflicting consciousness before and after to the attribute of the body percept: instinctive BSC (Stage 1) and objective object consciousness (Stage 4). The conflict involves whether the attribute of the body percept (reacted to by the brain) is instinctivity at Stage 1 or objectivity at Stage 4; how does the brain’s consciousness respond to this change in the attribute of percept? When the body percept attribute is recognized as objectivity at Stage 4, in the brain’s momentary reaction, the objectivity of the body percept suppresses instinctivity. Subsequently, the brain-generated objective object consciousness toward objectivity suppresses the instinctive BSC.^6^ Key here is the suppression placed by the non-restriction relationship between objectivity and the brain’s subjectivity on the restriction relationship between instinctivity and the brain’s subjectivity; the relationship between instinctivity and the brain’s subjectivity is also suppressed to be non-restricted. Just as instinctivity is “wrapped” by objectivity and its restrictive relationship with the brain’s subjectivity suddenly “torn off” by the non-restrictive relationship, the body’s instinctivity can no longer restrict the brain’s subjectivity. Thus, the brain experiences a dramatic change (Figure 1F). Here, the non-restricted brain suddenly realizes “the body (its own) was” “a completely independent individual” (see text footnote 1, respectively) (namely an objective “wrapping” (suppressing) instinctive individual) among its conspecifics. Toward the objectivity that suppresses instinctivity, as the attribute of its own body percept, this non-restricted brain generates a kind of BSC named objective BSC.

Hence, this stage’s CRP is expressed as: The brain, whose subjectivity is non-restricted with the objectivity that suppresses instinctivity, generates objective BSC toward the objectivity that suppresses instinctivity as the attribute of the body percept (Figure 1F). This stage is referred to as “brain–body detachment,” and the corresponding simplified CRF is expressed as:


Brain(subjectivity⊆objectivity⊇instinctivity)→Objectivity⊇instinctivity(body)=ObjectiveBSC (12)

Where “⊇” means “suppress”.

#### 4.2.6 Stage 6: subjective bodily self

In Stage 6, major changes are unstoppable. Once the non-restricted brain perceives the body’s instinctive impulse again, still being affected by instinctivity, it realizes the body is “a special individual” (see text footnote 1, respectively), namely that “the body” has always been the source of the brain perceiving, the reliance of the brain’s subjectivity, and the previous “self” in the “brain–body restricted” state. Moreover, since the brain enters a new state of “brain–body detachment,” the body percept possesses both instinctivity and objectivity. In turn, the brain attempts to randomly lift the arms and legs/direct the body; now, it can control the body and “self” image freely, like playing with a stone that objectively exists, instead of simply following the body’s directives as before. When the brain, in the new state of “brain–body detachment,” has a new reaction to both the instinctivity and objectivity of the body percept, the bodily self becomes a new, special percept whose novel attribute includes both instinctivity and objectivity, with objectivity suppressing instinctivity. This new attribute is a compound attribute, termed objectivity-suppressing-instinctivity. The new compound attribute of the bodily self still majorly influences the brain’s subjectivity through instinctivity but can no longer restrict subjectivity; meanwhile, subjectivity can influence and dominate the new bodily self attribute through subjective purpose and impulses (Figure 1G). Thus, the brain itself makes major progress: Instinct is no longer the only source of subjective purpose; the brain can create ideas on its own and freely lead the body to carry out these ideas. The brain surpasses the animal stage. Then, facing its body with the new compound attribute, the brain generates the first new consciousness of bodily self: This is “I” (see text footnote 1, respectively)—this new, complete, and independent conspecific individual is so special that the brain’s subjectivity has been depending on, expressing with, and acting for; this is “a special individual who could be freely controlled by itself” (see text footnote 1, respectively). Thus, self-recognition emerges; S/he (see text footnote 2) is “likely tempted to exclaim” “Ah!” (vocalization that approximates “I”) (see text footnote 1, 3, respectively). Thus, “I” is born (Figure 1G). The BSC in the brain at this new stage is called subjective BSC. The “I,” the first subjective BSC, can be considered the first consciousness.

This stage’s CRP is expressed as: The brain, whose subjectivity is non-restricted with the compound attribute of objectivity-suppressing-instinctivity, generates subjective BSC toward the compound attribute of the body percept (Figure 1G). This stage is referred to as “subjective bodily self.” The corresponding simplified CRF is expressed as:


Brain(subjectivity⊆(objectivity⊇instinctivity))→(Objectivity⊇instinctivity)(body)=SubjectiveBSC (13)

#### 4.2.7 Stage 7: subjective bodily metaconsciousness

Following the first consciousness, as the subjectivity of the brain is no longer restricted, the brain may form a new cognition of a specific attribute exhibited by the subjective bodily self referred to as “I” (see text footnote 1, respectively). This special attribute is in fact the manifestation of the brain’s subjectivity in the subjective bodily self—that is, some form of subjectivity, or rather subjective capacity, or cognitive capacity, expressed by “I.” In the thought experiment, this phenomenon is reflected in David’s thinking that “‘I’ can catch fish but cannot spot fish” (see text footnote 1, respectively), which demonstrates cognition of his own cognition, as well as behavior guided by this cognition: forming a partnership with Ruby to catch fish (Figure 1H). A large body of research on metacognition in non-human primates provides empirical support for this phenomenon (Beran, 2019). In a typical tubes task experiment (Call and Carpenter, 2001), bait was hidden inside one of four opaque horizontal tubes; human children, chimpanzees, and orangutans could choose to observe first before making a selection. When participants did not witness the hiding process, they preferred to observe first before deciding, indicating that they could evaluate their own cognitive state and adjust their behavior—thus demonstrating metacognitive ability. Metacognition refers to cognition about cognition, or thinking about thinking (Flavell, 1979; Smith et al., 2009). Its related concept, metaconsciousness, refers to consciousness of consciousness (Chin and Schooler, 2009). Within the framework of this study, metacognition is defined as the brain’s cognitive process toward some form of subjectivity; this cognitive process likewise gives rise to consciousness, which is defined as metaconsciousness. Accordingly, in the thought experiment, David’s ability to evaluate his own cognition and thereby adjust his behavior demonstrates his metacognition and metaconsciousness.

The subjectivity of the brain manifests as various subjective capacities, and bears an inclusive relationship with some form of subjective capacity or subjectivity. Hence, this stage’s CRP is expressed as: The brain, whose subjectivity includes some form of subjectivity, generates subjective bodily metaconsciousness toward some form of subjectivity of the bodily self percept (Figure 1H). This stage is referred to as “subjective bodily metaconsciousness.” The corresponding simplified CRF is expressed as:


$$\begin{array}{l} \text{Brain}\;(\text{subjectivity}⊇(\text{some form of subjectivity}))\to \\ (\text{Some form of subjectivity})\;(\text{bodily self})=\\ \text{Subjective bodily metaconsciousness} \end{array}$$ (14)

Where “⊇” means “include”.

#### 4.2.8 Stage 8: subjective mental self

The emergence of subjective bodily metaconsciousness greatly enhances the brain’s cognitive capacity and level of consciousness. When individuals gain rewards or competitive advantages through the application of metacognitive abilities (Call and Carpenter, 2001; Hare et al., 2000), it may motivate related behaviors. Accordingly, in the thought experiment, David’s application of metacognition is motivated: for instance, through continuous evaluation, comparison, application, and reflection on the metacognition of himself and other conspecifics, he promotes his brain’s learning of metacognitive abilities and the scope development of corresponding metaconsciousness. David evaluates that “Jimmy can do this but not that; Kitty can do that but not this; ‘I’ cannot do those things but can do these” (see text footnote 1, respectively), and so on, and increases his own metacognitive ability items by adjusting his behavior to cultivate diverse partnerships (Figure 1I left). In other words, the brain continuously develops a metacognitive “map” of the “I,” thereby constantly enriching the connotation of the “I”‘s metaconsciousness—analogous to creating an increasingly rich and clear “portrait” of the various forms of subjectivity of the “I.” The brain keeps evaluating the “I,” attempting to ask and answer: What can “I” do? … “What else can ‘I’ do?” (see text footnote 1, respectively) (Figure 1I left) until 1 day, the brain inevitably poses the first fundamental question: “Who am ‘I’?” (see text footnote 1, respectively) At this moment, the brain regards its own overall subjectivity—including all forms of subjectivity (and their states associated with memory)—as a single cognitive object and reacts to it. The brain develops a conscious reaction to itself; while the brain itself becomes the object of perception (Figure 1I right). As soon as the brain perceives only itself, its subjectivity completely eliminates the influence of the body’s instinctivity and outside objects’ objectivity and thus becomes a completely independent attribute of the brain. The reaction of the brain (whose subjectivity is completely independent) to the independent subjectivity of the brain percept then becomes a completely free and independent consciousness of the brain; MSC and the mental self are formed simultaneously (Figure 1I right).

When the brain asks “Who am ‘I’?” it begins to observe its bodily self and mental self with MSC in a new light. Thus, the mental and bodily selves, “the brain and body,” are reunited. The first answer is “‘I’ am a MAN!” Thus, “I” is defined, and “MAN,” in its true meaning—human—is come to be.

This stage’s CRP is expressed as: The brain, whose subjectivity is independent, generates independent and subjective MSC toward the independent subjectivity of the brain percept (Figure 1I right). This stage is referred to as “subjective mental self”. The corresponding simplified CRF is expressed as:


$$\begin{array}{l} \text{Brain}\;(\text{subjectivity independent})\to \text{Independent}\\ \text{subjectivity}(\text{brain})=\text{Independent and}\\ \text{subjective}\;\mathrm{MSC} \end{array}$$ (15)

### 4.3 Functional formulation of the model

#### 4.3.1 MLC model

In this way, with the definition of consciousness as the theoretical basis and the CRF as the underlying formula, a comprehensive MSC origination model is constructed, comprising eight continuous and changing CRFs per the phenomena, process, and result of the Lakeside Image thought experiment. This model is termed the MLC model (see Table 1). It adequately and theoretically explains the MLC process underlying the origination of human MSC and is supported experimentally by the RHI.

**Table 1:** The model of the Mirror-Induced Leap of Consciousness (MLC) process for the origination of mental self-consciousness (MSC) (Decomposed): eight stages of continuously changing conscious reaction process.

Stage	Name	Subject of consciousness	Object of consciousness	Simplified CRF* including the outcome of consciousness
1	Brain–body restricted	The brain, whose subjectivity is restricted by instinctivity	The instinctivity of the body percept	Brain (subjectivity ⊆ instinctivity) → Instinctivity (body) = Instinctive BSC
2	Brain–image non-restricted	The brain, whose subjectivity is non-restricted with objectivity	The objectivity of the image percept	Brain (subjectivity ⊈ objectivity) → Objectivity (image) = Objective object consciousness
3	Image–body identity	The brain, whose subjectivity is restricted by instinctivity	The instinctivity of the image percept that is subjectively identified with the body percept	Brain (subjectivity ⊆ instinctivity) → Instinctivity (image = body) = Instinctive BSC
4	Body–image objectivity	The brain, whose subjectivity is non-restricted with objectivity	The objectivity of the body percept that is subjectively identified with the image percept	Brain (subjectivity ⊈ objectivity) → Objectivity (body = image) = Objective object consciousness
5	Brain–body detachment	The brain, whose subjectivity is non-restricted with the objectivity that suppresses instinctivity	The objectivity that suppresses instinctivity as the attribute of the body percept	Brain (subjectivity ⊈ objectivity ⊇ instinctivity) → Objectivity ⊇ instinctivity (body) = Objective BSC
6	Subjective bodily self	The brain, whose subjectivity is non-restricted with the compound attribute of objectivity-suppressing-instinctivity	The body percept’s compound attribute of objectivity-suppressing-instinctivity	Brain (subjectivity ⊈ (objectivity ⊇ instinctivity)) → (Objectivity ⊇ instinctivity) (body) = Subjective BSC
7	Subjective bodily metaconsciousness	The brain, whose subjectivity includes some form of subjectivity	Some form of subjectivity of the bodily self percept	Brain (subjectivity ⊇ (some form of subjectivity)) → (Some form of subjectivity) (bodily self) = Subjective bodily metaconsciousness
8	Subjective mental self	The brain, whose subjectivity is independent	The independent subjectivity of the brain percept	Brain (subjectivity independent) → Independent subjectivity (brain) = Independent and subjective MSC

*See Section 4.2. for details.

#### 4.3.2 Formalized functional expressions

What is the intrinsic mechanism underlying the MLC model and the conscious changes it reflects? This can be elucidated by formalizing the transformations of CRPs and their corresponding CRFs as functional expressions.

##### 4.3.2.1 Foundational definitions

1 Let *p* ∈*P* denote a basic percept, constituting the Set of Basic Percepts, *P* = {*B*, *O*, *S*}, where:
  - *B*: Body (bodily self)
  - *O*: Outside Object
  - *S*: “Self” — specifically referring to the Brain Itself
Let *a* ∈ *A* denote an attribute of *p*, constituting the Set of Basic Attributes, *A* = {*Ins*, *Obj*, *Sub*}, where:
  - *Ins*: Instinctivity
  - *Obj*: Objectivity
  - *Sub*: Subjectivity
If a percept *p* possesses an attribute *a*, it is denoted as *a(p)*. Consequently, the fundamental attributes of the three basic percepts are:
  - *B* is fundamentally characterized by *Ins*, denoted as *Ins(B)*.
  - *O* is fundamentally characterized by *Obj*, denoted as *Obj(O)*.
  - *S* is fundamentally characterized by *Sub*, denoted as *Sub(S)*.

##### 4.3.2.2 Core formulas

Building upon the definition of consciousness and drawing from the analysis of changes in CRFs within the MLC model, it can be posited that changes in consciousness *C* fundamentally reflect alterations in a specific cognitive relation or constraint between the brain’s subjectivity *Sub* and the attribute of percept *a(p)*. I abstract this variable relation as the Consciousness Cognitive Relational Function *R*, which represents the interaction between the brain’s subjectivity and the attribute of percept in the conscious cognitive process. Mathematically, consciousness is then the image mapped by the brain’s subjectivity *Sub* through the Consciousness Cognitive Relational Function *R* acting upon the attribute of percept *a(p)*. Thus, the CRF can be simplified into the Consciousness Cognitive Relational Function:


C=R(a(p)) (16)

To formalize percept drift and its resultant attribute drift effect, we introduce the percept drift function, PD:


$$\mathrm{PD}\;(a_{1}(p_{1}),a_{2}(p_{2}))=a_{1}(p_{2}=p_{1})$$ (17)

*Interpretation*: This function describes the effect of percept drift. Due to the synchronous connection established between p₂ and p₁ through repetitive stimulation, the attribute a₁ associated with percept p₁ is transferred or mapped onto percept p₂. Consequently, percept p₂ is experienced as possessing attribute a₁ (the attribute originally belonging to p₁), and p₂ is subjectively identified as p₁. This outcome is denoted in the function as *a₁(p₂ = p₁)*. For a detailed explanation, see Section 4.2.3.

##### 4.3.2.3 Functional representation of the MLC process

Applying the functions to express the MLC yields Table 2.

**Table 2:** Functional formalization of the MLC process for MSC origination.

Stage	Functional expression of consciousness	Cognitive relational changes driven by variables of attribute of percept	Corresponding conscious phenomenon	Explanation of conscious change
1	*C₁ = R(Ins(B))*	*Sub* ⊆ *Ins*	Instinctive BSC	The normal state of BSC in the animal stage.
2	*C₂ = R(Obj(O))*	*Sub* ⊈ *Obj*	Objective object consciousness	The normal state of object consciousness in the animal stage.
3	*PD (Ins(B), Obj(O)) = Ins (O=B)* *C₃ = R(Ins(O=B))*	*Sub* ⊆ *Ins*	Instinctive BSC	Repetitive mirror stimulation triggers percept drift, producing the attribute drift effect, which transforms the brain’s objective object consciousness of the image into instinctive BSC.
4	*PD (Obj(O), Ins(B)) = Obj (B = O)* *C₄ = R(Obj(B=O))*	*Sub* ⊈ *Obj*	Objective object consciousness	A reverse percept drift occurs, generating objective object consciousness toward one’s own body.
5	*C₅ = R(Obj* ⊇ *Ins (B))*	*Sub* ⊈ *Obj* ⊇ *Ins*	Objective BSC	Conflict arises from the dual attributes of the body percept (*Obj(B)* from Stage 4 vs. *Ins(B)* from Stage 1). Objectivity “wraps” instinctivity, “tearing off” (suppressing) its restrictive relation with subjectivity. The brain generates objective BSC toward the body.
6	*C₆ = R((Obj* ⊇ *Ins)(B))*	*Sub* ⊈ (*Obj* ⊇ *Ins*)	Subjective BSC	Subjectivity, no longer restricted by instinctivity, generates subjective BSC toward the body’s new compound attribute of objectivity-suppressing-instinctivity, “This is ‘I’.” Self-recognition emerges.
7	C₇ = R(SSub* ⊈ *(Obj* ⊇ *Ins)(B^†^))*	*Sub* ⊇ *SSub* ⊈ (*Obj* ⊇ *Ins*)	Subjective bodily metaconsciousness	The brain generates metaconsciousness toward bodily self’s some form of subjectivity that remains unrestricted by the compound attribute.
8	*C₈ = R(Sub(S))*	*Sub* independent	Independent and subjective mental self-consciousness (MSC)	The accumulation of metaconsciousness toward multiple singular subjectivities of the bodily self ultimately undergoes a qualitative leap, giving rise to the question “Who am ‘I’?”. This marks the genesis of independent and subjective MSC.

For explanations of the attributes relational operators (⊆, ⊈, ⊇), see the Glossary or Section 4.2; SSub*, some form of subjectivity; B^†^, bodily self.

As demonstrated in Table 2, in the MLC process, the Percept Drift Function causes the brain-perceived attribute of the body percept to shift from instinctivity to objectivity. This triggers a cascade of changes in the cognitive relation between the brain’s subjectivity and the perceptual attribute, as well as in the values of the Consciousness Cognitive Relational Function, ultimately leading to the complete independence of the brain’s subjectivity. This independent subjectivity then becomes the object of the brain’s own cognition, realizing the genesis of MSC.

## 5 Conclusions and discussion

### 5.1 The origin of self-consciousness

This study attributes the origin of human self-consciousness to the origination process of mental self-consciousness (MSC). It proposes the Mirror-Induced Origination Hypothesis of human MSC and conducts the Lakeside Image thought experiment to test this [see Section 3.1. (Figure 1)]. The model of the Mirror-Induced Leap of Consciousness (MLC) process for the origination of MSC is constructed (see Table 1), and the MSC origination process is explained stage by stage through a detailed analysis of the eight CRPs, CRFs, and their transitions in the model (see Section 4.2.). Based on the participant’s experimental phenomena, these eight CRPs are continuous but different. Figure 1, Tables 1, 2 and the theoretical analysis indicate the changes in these CRPs are driven by a cascade of changes in the multiple variables within the CRF (“percept,” “attribute of percept,” “consciousness,” and even “brain,” see Equation 1) and their interrelations—particularly the cognitive relation between the brain’s subjectivity and the attribute of percept. Among these, percept drift induced by repetitive mirror stimulation serves as the primary trigger for the key transformations. Most importantly, in this experiment, the conscious reaction’s change at each stage alters/increases the brain’s conscious level, precipitating a continuous eight-stage ladder-like jumping process (leap) of the brain’s conscious reaction throughout the experiment. Finally, the brain generates the conscious reaction directly to the brain percept with attribute of independent subjectivity, and MSC emerges (the mental self emerges simultaneously; see Figure 1I). Roughly expressed as CRF, it is:


Brain→Attribute of brain=MSC (18)

Accurately expressed as CRF, it is Equation 15 (see Section 4.2.8., or Table 1 for a detailed explanation). Mathematically expressed as Consciousness Cognitive Relational Function, it is:


MSC=R(Sub(S)) (19)

Based on the theoretical analysis, explanation of the process, and thought experiment results, human MSC’s origination can be considered a Mirror-Induced Leap of Consciousness (MLC) process during the evolutionary course of consciousness wherein the conscious reaction in humans’ animal ancestors’ brain was induced to undergo illusory transformation and continuous leap-like changes under the repetitive stimulation of the mirror image of its own body. Here, the brain’s conscious level jumped continuously and simultaneously; finally, the brain’s conscious reaction turned into the conscious reaction of the brain to the brain, thus forming MSC. This conclusion supports the Mirror-Induced Origination Hypothesis of human MSC.

The eight-stage MLC process may appear as a dramatic moment in the brain, but it in fact represents the awakening and liberation of consciousness achieved by our ancestors over millions or even tens of millions of years of evolution. The expression of the MLC process in the form of the Consciousness Cognitive Relational Function actually represents the evolutionary mechanism of consciousness, the essence of which lies in the liberation of the brain’s subjectivity from the constraints of the body’s instinctivity. The course of conscious evolution is precisely the process through which the brain’s subjectivity progressively frees itself from these constraints, seeks independence and freedom, and thereby continuously evolves.

According to its genesis process, MSC is defined as the brain’s reaction to its independent subjectivity, including the state of the independent subjectivity. Meanwhile, as per Equations 7, 15, 18, 19, and the definition of percept, the mental self is defined as the outcome of the brain perceiving itself (or the functional brain acting as a percept). These all constitute the outcomes of the evolution of consciousness.

The MLC model offers a comprehensive logical deduction, theoretical analysis and explanation for the genesis of the most crucial MSC during the evolutionary course of consciousness at the brain cognitive level. According to the Lakeside Image thought experiment and the interpretation of the MLC model, human MSC may have ultimately formed as the outcome of continuous changes in conscious states and levels, induced during evolution by the accidental repetitive stimulation of one’s own mirror image. In other words, human self-consciousness may have been reasonably generated through natural evolution, thereby providing a cognitive-level explanation for the “hard problem” of consciousness, as well as a possible answer to the eternal philosophical questions: Who am I? Where did we come from? From God or ourselves (Eccles, 1989, p. xii, 249)?

The origin of self-consciousness is the fundamental inception of human beings, an evolutionary miracle, and this study’s results reasonably infer its emergence. Empirical evidence at the individual behavioral level, derived from experiments on modern humans, chimpanzees, rhesus macaques, and other species—including the standard mirror test, extended mirror test, RHI experiment, and tubes task experiment—provides indirect support at the brain cognitive level for this emergence. However, the Lakeside Image thought experiment, which posits how human ancestor acquired MSC, remains difficult to confirm directly through real experimental case.

Moreover, the implication of this study—that self-consciousness originated through natural evolution—is not limited to humans but extends to all life in the universe. As per the MLC model and its conclusions, HPT animals such as chimpanzees can reach Stage 6 of MLC (subjective bodily self, see Section 4.2.6.; Figure 1G), possessing subjective BSC (the first consciousness of “I,” see Section 4.2.6.) and self-recognition of body. Related questions then arise: When did they acquire self-recognition? Do they (or some of them) possess MSC? Was the evolution from self-recognition to MSC gradual or saltatory? Taking chimpanzees as an example, they share a last common ancestor with humans and rhesus macaques approximately 25 million years ago (Kumar and Hedges, 1998), and a last common ancestor solely with humans approximately 6 million years ago (Patterson et al., 2006). Thus, the acquisition of self-recognition in chimpanzees could have occurred either in the common ancestor era with humans (i.e., between approximately 23 and 6 million years ago) or after divergence. If we can demonstrate that modern chimpanzees do not possess MSC, it would be reasonable to infer that humans acquired MSC independently after diverging from chimpanzees, within approximately 6 million years, and possibly through gradual evolution. Conversely, if chimpanzees do possess MSC, it could be explained by inheritance from a common ancestor or by independent acquisition after divergence, without ruling out the possibility of saltatory evolution—It should be noted that the “leap-like” changes proposed in this study refer primarily to leaps in logical and conceptual deduction, as well as in the level of consciousness; they are not equivalent to biological mutations, nor do they exclude the possibility of gradual evolution (e.g., the cumulative acquisition of metacognitive capacities across Stages 7–8). Therefore, this issue holds significant importance: How can we experimentally test whether chimpanzees possess MSC? If traditional animal cognitive behavioral experiments, such as the mirror test that chimpanzees already pass, could be redesigned with reference to MLC Stages 6–8 (see Section 4.2.6–8.; Figures 1G–I) to test bodily metaconsciousness and MSC in HPT animals, remarkable new discoveries are possible. Here, animals with MSC are considered “intelligent animals” and life forms with MSC “intelligent life.” I believe that chimpanzees and some other HPT animals (e.g., elephants, dolphins), as life partners of the human species, are likely intelligent animals possessing MSC, similar to humans. If this could be proven, it would raise new ethical issues and research horizons.

For MFT non-human primates, the extended mirror test with training (Chang et al., 2015) must be extensively replicated and verified. On this basis, further experiments should be designed to test whether such MFT animals, as well as those HPT animals that may be verified as lacking MSC, can acquire bodily metaconsciousness and MSC through artificial interventions such as induction by repetitive mirror stimulation—that is, whether humans can intervene in natural evolution, and whether humans can play a role analogous to “God” (Eccles, 1989, p. xii, 249). For non-visually dominant animals, their possible mode of self-recognition may differ from “mirror self-recognition”; that is, they may not need to achieve self-recognition through visual means. Corresponding non-visual self-recognition testing methods and induction conditions merit exploration.

### 5.2 Cognitive theory framework

This study restructures a consciousness-based cognitive theory framework. By redefining foundational concepts, it establishes a coherent conceptual system in cognitive science that traces from sensation, perception, and percept to consciousness, bodily self-consciousness (BSC), and mental self-consciousness (MSC) (see the Glossary). Grounded in the definition of consciousness as the brain’s reaction to the attribute of a percept and the corresponding Conscious Reaction Formula (CRF), the framework elucidates the core mechanisms of conscious change, conscious evolution, and the origin of self-consciousness through the Consciousness Cognitive Relational Function (*C = R(a(p))*), which represents the interactive relationship between the brain’s subjectivity and the attribute of a percept. Centered on this foundation, the framework integrates key hypotheses (the Mirror-Induced Origination Hypothesis and Hypotheses 1–3), experimental reproduction (the Lakeside Image thought experiment, Figure 1), logical reasoning (the eight-stage deductive process, Equations 1–19), core mechanisms and principles (e.g., the function *R*, percept drift), empirical evidence (e.g., the rubber hand illusion (RHI), mirror test, and metacognition experiments), formal modeling (Table 1), and mathematization (Table 2), thereby developing a novel comprehensive cognitive theory framework: the Mental Self-Consciousness Cognitive Theory (MCT) framework.

MCT is also a consciousness theory at the cognitive level. Compared with mainstream consciousness theories, it adopts a non-traditional research pathway while maintaining multiple connections with them, as detailed in the comparative analysis in Table 3. The contrast in Table 3 shows that traditional mainstream consciousness theories (GNW, HOT, IIT) represent in-depth investigations of specific aspects of consciousness employing distinct paradigms. GNW focuses on general conscious processes; MCT builds on the general process to explore consciousness at various levels, further investigating conscious changes and focusing on the mirror-induced origination of MSC. HOT centers on the subject-object relationship of consciousness; MCT explains this relationship and its changes through the interaction of the three elements of consciousness, encompassing the metacognitive relationship emphasized by HOT and highlighting the principle of percept drift. IIT focuses on the high-level quantification of general conscious outcomes; MCT transforms subjectively experienced consciousness into objectively describable variable relationships, proposing formulation of consciousness at different levels and functionalization of conscious changes. In summary, MCT can be regarded as a systematically innovative theory of consciousness that focuses on explaining the origin of self-consciousness from an evolutionary perspective.

**Table 3:** Comparison between mainstream consciousness theories and MCT.

Comparison dimension	Global Neuronal Workspace (GNW)	Higher-Order Thought theory (HOT)	Integrated Information Theory (IIT)	Present study (MCT)
Research level	Neural mechanism level	Brain cognitive level	Individual experiential level	Brain cognitive level
Definition of consciousness	Information that is coordinately activated and widely broadcast in the brain-wide “workspace” by distributed neuronal populations	Higher-order thoughts about lower-order mental states	Maximally Irreducible Conceptual Structure (MICS) – an irreducible constellation of concepts	The brain’s reaction to the attribute of percept (MSC refers to the brain’s reaction to its own independent subjectivity)
Core goal (strength)	To explain the processing mechanism of consciousness	To explain the metarepresentational nature of consciousness	To quantify the level and quality of consciousness	To explain the generation and change processes of consciousness
Core mechanism	Attentional amplification + Global broadcasting	Metarepresentation (Higher-Order Thought)	Causal integration (generating the Maximally Irreducible Conceptual Structure, MICS)	Consciousness Cognitive Relational Function *R(a(p))* + percept drift + Mirror-Induced Leap of Consciousness (MLC)
Key empirical evidence	Masked priming, binocular rivalry, P300 component	Blindsight, verbal reports, subliminal emotional processing	Changes in Φ ^max^ value under anesthesia, unconscious contribution of the cerebellum, Φ ^max^ calculation in simple systems	Mirror test, monkey mirror training, Lakeside Image thought experiment, rubber hand illusion (RHI), tubes task experiment
Focused conscious aspect	Conscious processes	Conscious subject-object relationships	Conscious content and outcomes	Conscious processes and outcomes, the attribute relationships and changes of conscious elements, changes in multiple conscious processes
Primary focus	Neural processing mechanism	Relational nature	Content quantification	Conscious evolution and the origination of MSC
Connections with the present study	MCT applies the mechanism of multisensory integration to support the definition of perception, associating with the global broadcasting of distributed neurons in GNW; the selection of percept by attention in MCT is related to the attentional amplification mechanism of GNW. Both theories investigate conscious processes: MCT focuses more on mesoscopic cognitive mechanisms, while GNW emphasizes microscopic neural mechanisms, enabling mutual complementarity.	MCT includes the subjective bodily metaconsciousness stage of MLC, associating with the higher-order thoughts in HOT; the reflective characteristic of MSC is an extension of metarepresentation; the lower-order mental states in HOT can be linked to the low-stage consciousness and perceptual cognition in MLC. Both theories explore cognitive processes and levels, with MCT being more systematic and coherent.	The changes in consciousness levels across MLC stages in MCT can be associated with the application of Φ value quantification in IIT; the concept of MICS in IIT is related to the concept of percept and the conscious cognition of percept in MCT. IIT tends to vertically abstract consciousness from the macroscopic experiential level to the microscopic neural mechanism level through quantitative measurement, thereby seeking relevant evidence and providing explanations; in contrast, MCT tends to adopt a horizontal approach to analyzing cognitive processes at the mesoscopic cognitive level and seeks existing and potential evidential support at the neural level.	–

Given the developmental challenges faced by cognitive science in understanding consciousness, MCT provides systematic innovations for restructuring a complete and unified consciousness-based cognitive theory; see Table 4 for detailed explanations.

**Table 4:** Systematic innovations of Mental Self-Consciousness Cognitive Theory framework for restructuring consciousness-based cognitive theory.

No.	Innovative idea
1	Clarifying the vague and overlapping foundational concepts of sensation, perception, percept, and consciousness in traditional cognitive theory laid the cornerstones for logically restructuring the concept system of cognitive theory
2	Defining consciousness as a generative process transformed individual subjective experiences into objective attributes of the brain and the percept as well as their relations, and formalized them, addressing the “hard problem” that subjective experiences are difficult to objectively explain or “arise from physical systems” (Chalmers, 1996, p. ix, 5, 107), laying the “keystone” for restructuring cognitive theory
3	Defining MSC as per its origination process led to the construction of the “dome” of cognitive theory
4	Proposing a formalized, functional model of consciousness using CRF, *R(a(p))* function and the MLC framework provided a sample for the integrative approaches of consciousness theory research (Signorelli et al., 2021)
5	Constructing a logical system of cognitive concepts based on CRF; defining the relationships among MSC, BSC, and traditional self-consciousness; and building an integrated concept system from sensation, percept, consciousness, and BSC to MSC provided a framework for restructuring cognitive theory
6	Although limited by the length of the study, the expansion of the core concept system of cognitive theory is implied. Ideas such as instinct, memory, experience, learning, and thinking can be conceptually associated based on the generation and definition of consciousness
7	A series of hypotheses, premises, principles, and mechanisms explained experiments and experiences based on the concept system and became the pillars of cognitive theory with explanatory power
8	Proposing three essential attributes and their interactions for basic existences revealed the intrinsic mechanism of the transformations and evolution of consciousness and may inspire cognitive theory, neural mechanisms, genetic science, and evolutionary studies
9	The successful application of the theory, including the percept drift principle, in explaining behavior and cognition implies its potential value in corresponding research on microscopic neural mechanisms
10	Presenting the possibility of constructing a cognitive theoretical system unified across behavioral, cognitive, and neural levels

MCT provides an explanatory framework that unifies the behavioral, cognitive, and neural levels. Core MCT concepts (perception, percept, consciousness, BSC, and MSC) can effectively describe basic cognitive processes and their interrelated mechanisms in the brain, which preliminarily achieves a consistency among the core concept system of cognitive theory, experimental phenomena, and subjective experiences. MCT successfully explains, interprets, and correlates typical cognitive behavioral experiments such as the RHI, mirror test, extended mirror test, and tubes task, demonstrating MCT’s explanatory power, practical value, and important implications. It provides a comprehensive, empirically supported theoretical explanation for the origin of human self-consciousness. The percept drift principle in MCT emphasizes the significance of repetitive mirror stimulation in inducing illusory transformation and consciousness changes experimentally and theoretically. MCT thus accounts for the shared key cognitive process underlying both RHI and MSC’s origination. Advances in micro-level research on neural mechanisms directly support these conclusions. The neuronal mechanism integrating multisensory stimulation (Andersen, 1997; Blanke, 2012; Blanke et al., 2015) and the feature detectors discerning distinct features of a single sensory stimulus (Kandel and Squire, 2000; Zimbardo et al., 2014) form the physiological basis of percept, consciousness, and BSC at the meso brain cognitive level. Systematic and long-term multisensory stimulation may lead to neuronal changes and establish new neural connection mechanisms (Blanke et al., 2015; Graziano et al., 2000), providing possibilities for consciousness evolution and MSC generation; by contrast, the percept drift principle within the MCT framework provides a meso-level guidance to study these possible neural connection mechanisms and neuronal changes. Further research must clarify how these micro-level neural mechanisms precipitate the brain’s various meso-level functions. Here, the neural mechanisms of diverse brain reactions—including object consciousness, BSC, and MSC—must be systematically studied, compared, and verified based on the integration mechanisms of multisensory stimulation. The “binding problem” of how the brain combines the multiple features from feature detectors into a single percept remains unknown (Kandel and Squire, 2000; Zimbardo et al., 2014).

I believe that cognitive psychology and cognitive neuroscience describe the same cognitive processes at different levels, but they currently remain on opposite sides of the same mountain, hence the need to meet halfway. While the neural mechanisms of consciousness have yet to be fully explained (Aru et al., 2012), most major advances in the neural basis of behavior over the past century have relied on the principles of cognition for guidance (Frank and Badre, 2015). On a broader scale, the three domains of biological neural mechanism, artificial neural mechanism, and cognitive theory can inspire and benefit each other and achieve long-term development (Hoffmann et al., 2021; De Croon et al., 2022). Therefore, the findings of this study on the advancement of consciousness theory and cognitive theory may provide insights for both biological neural mechanism research and artificial neural mechanism development. Particularly, the Conscious Reaction Formula (CRF), Consciousness Cognitive Relational Function (*R(a(p))*), Percept Drift Function (*PD*), and Mirror-Induced Leap of Consciousness (MLC) model under the MCT framework develop an original brain-inspired mathematical model of consciousness generation and change that can be applied to neural and evolutionary computing. Considering the possibility of the natural evolution of MSC in the context of MCT, combined with the exploration and development of relevant experiments and research (Krauss and Maier, 2020), the creation of “machine consciousness” is not impossible.

As a new cognitive theory framework, MCT must be consolidated and strengthened both theoretically and empirically. On the one hand, its concepts need to be extended, its cognitive principles enriched, and its mathematical formalization deepened. On the other hand, broader explanations and verifiable predictions at the cognitive, behavioral, and neural levels are required, so as to achieve successful manipulation and effective research guidance.

Finally, building a unified cognitive theoretical system across multiple levels requires integrating traditional methods with the latest technologies [e.g., digital technology, AI, VR, augmented reality, brain-computer interfaces (Kline et al., 2021), neuroimaging] to understand the correlations among “machine consciousness,” human behavior, brain cognition and consciousness, and neural mechanisms. This will open new research spaces in the philosophy of the mind (Gallagher, 2000), computer science, ethology, psychology (Dresp-Langley, 2021), cognitive science, and neuroscience. Thus, a clearer outline of the “huge mystery” can be developed from the internal correlation mechanisms and external representations at the macro, meso, and micro levels. In the spatial–temporal context of consciousness’ evolution and MSC genesis, a new understanding is gained of David Hume’s (2000) dictum “the self is nothing but a bundle of perceptions” and Descartes (2016) “I think, therefore I am”.

## Data Availability

The original contributions presented in the study are included in the article/supplementary material, further inquiries can be directed to the corresponding author/s.

## Footnotes

1 The statements of the thought experiment are quoted sentence by sentence in the following sections and are analyzed and explained in detail.

2 With the emergence of self-recognition, the pronoun “S/he” is used to refer to the human animal ancestor instead of “It.”

3 As a natural human vocalization, “Ah” is similar to the vocalization of “I” in English. Similarly, the vocalization of “I” in Chinese is similar to the natural human vocalization of “wow.” Perhaps there are similar characteristics in other languages.

4 The statements of this definition are cited in the following section.

5 One clear piece of evidence is that, in the evolutionary history from lower to higher animals, the brain and nervous system gradually diverged from the body, both organizationally and functionally. Understood from the perspective of the evolution of consciousness in this study, this suggests a relationship between the subjectivity of the brain and the instinctivity of the body percept.

6 In fact, it is owing to this that human beings can overcome their instincts and carry out dangerous behaviors such as trying to detect fire with their hands.

## Glossary

I. Basic cognitive processes
Sensation: Standard term: the process by which the sensory receptors and nervous system receive and represent stimulus energies from the environment. The foundation of the concept system in this study (Goldstein and Cacciamani, 2022).
Perception: The process by which the brain recognizes and organizes sensations (to form a *percept*) (this study).
Percept: The product of perception; the combination of specific sensations recognized and formed from sensations received from environmental stimuli—the brain’s Processing Object Unit (POU) for sensations. Serves as the object of conscious reaction (this study).
Attention: The brain’s selection of percept—a definition consistent with the standard theoretical interpretation of selective information processing in the field of cognitive science but specifically situated within the perception-percept-consciousness conceptual chain of the present study (this study; cf. Eysenck and Keane, 2015; Pashler, 1998).
Consciousness: The core definition: the brain’s reaction to the attribute of percept, including the state of this attribute (this study).
Conscious reaction process (CRP): The process by which the brain generates consciousness (this study).
Conscious reaction formula (CRF): The basic formula for the CRP: Brain → Attribute of Percept = Consciousness (this study).
II. Core constructs
Brain: The subject of consciousness; the brain as cognitive function; one of the three kinds of basic existence (basic percept) (this study).
Body: The body that the brain inhabits; one of the three kinds of basic existence (basic percept) (this study).
Outside object: The object outside one’s body; one of the three kinds of basic existence (basic percept) (this study).
Bodily self: The percept of the body formed by the brain recognizing and organizing bodily sensations (this study).
Mental self: The outcome of the brain perceiving itself (or the functional brain acting as a percept); the additional connotation of the traditional “self” beyond the bodily self, essential for identifying and recognizing the “self” (this study).
Objectivity (Obj): The attribute of external, objective existence. Fundamental attribute of the outside-object percept (this study).
Instinctivity (Ins): The attribute of innate, biological existence. Fundamental attribute of the body percept (bodily self) (this study).
Subjectivity (Sub): The attribute of self-generation and initiative. Fundamental attribute of the brain (mental self); the source of consciousness (this study).
Object Consciousness: The brain’s reaction to the attribute of the outside object, including the attribute’s state (this study).
Bodily Self-Consciousness (BSC): The brain’s reaction to the attribute of the body it inhabits, including the state of this attribute (this study).
Mental Self-Consciousness (MSC): The brain’s reaction to its own independent subjectivity, including the state of the independent subjectivity (this study).
III. Key hypothesis, principles and framework
Mirror-induced origination hypothesis: The central hypothesis: humans’ animal ancestor was induced by repetitive stimulation of its own mirror image to eventually accomplish the crucial leap of “self-recognition,” leading to the emergence of MSC (this study).
Percept drift: The core cognitive principle: the process where the brain subjectively identifies different percepts as the same owing to the synchronous connection triggered by similarity between sensations from stimuli (such as vision and motion) derived from perceptual objects and the brain’s memory and experiences regarding sensations forming certain percepts (this study).
Attribute drift effect: The phenomenological consequence of percept drift: the brain experiences percept B as possessing attributes of percept A, as if the attributes of percept A had drifted to percept B (this study).
Mirror-induced leap of consciousness (MLC): The proposed eight-stage process for the genesis of MSC during the evolution of consciousness wherein the conscious reaction in the brain of humans’ animal ancestor was induced by repetitive mirror-image stimulation to undergo an illusory transformation and continuous, leap-like changes; during this process, the brain’s conscious level underwent simultaneous leaps, ultimately culminating in the brain’s conscious reaction turning back upon itself (this study).
Mental Self-Consciousness Cognitive Theory (MCT) framework: A consciousness-based cognitive framework proposed in this study that explains the origin of self-consciousness from an evolutionary perspective. Centered on the definition of consciousness as the brain’s reaction to the attribute of a percept, MCT redefines core cognitive concepts including perception, percept, BSC, and MSC. Built around the MLC process, the framework integrates hypotheses, experiments, logical justification, core principles, empirical evidence, modeling, and mathematization into a coherent theoretical system. It proposes that MSC emerged in the brain of humans’ animal ancestor through repetitive mirror-image stimulation and the pivotal transformation of percept drift, and explains typical cognitive phenomena and experiments (this study).
IV. Model and formalism
MLC model: A mathematical model that abstractly describes and explains the MLC process underlying the genesis of MSC. Built upon the definition of consciousness as its theoretical foundation and the CRF as its basic formalism, the model comprises eight successively transformed CRFs corresponding to the eight stages of the MLC process. It reveals the intrinsic mechanisms of conscious evolution through the interactions and changes of variables within the CRFs (this study).
Set of basic percepts (*P*): *p* ∈*P*, *P* = {*B*, *O*, *S*}, where *B* = body percept, *O* = outside object percept, *S* = brain itself (this study).
Set of basic attributes (*A*): *a* ∈ *A*, *A* = {*Ins*, *Obj*, *Sub*}, where *Ins* = instinctivity, *Obj* = objectivity, *Sub* = subjectivity (this study).
Consciousness cognitive relational function (*R*): A function representing the interaction between the brain’s subjectivity and the attribute of percept in the conscious cognitive process. (this study).
Consciousness cognitive relational functional expression (*C* = *R*(*a*(*p*))): The simplified representation of the CRF expressed in the form of an *R* function, where C denotes consciousness, *R* denotes the Consciousness Cognitive Relational Function, and *a*(*p*) denotes the attribute of percept *p* (this study).
Percept drift function (*PD*): A function describing the effect of percept drift: *PD* (*a₁*(*p₁*), *a₂*(*p₂*)) = *a₁*(*p₂* = *p₁*). Due to percept drift, the attribute *a₁* associated with percept *p₁* is transferred or mapped onto percept *p₂*, percept *p₂* is experienced as possessing attribute *a₁*, and *p₂* is subjectively identified as *p₁* (this study).
Attributes relational operators (⊆, ⊈, ⊇): The specific manifestations of the interaction mechanisms among the variables within the R function and the CRF. *Sub* ⊆ *Ins*: subjectivity restricted by instinctivity; *Sub* ⊈ *Obj*: subjectivity non-restricted with objectivity; *Obj* ⊇ *Ins*: objectivity suppresses instinctivity; *Sub* ⊇ *SSub*: subjectivity includes some form of subjectivity (this study).
V. Experiments and empirical evidence
Animal crypsis illusion: A perceptual phenomenon wherein animals blend into their environment through mimicking features of the environment to go undetected by predators. The cryptic animals apply Gestalt principles such as figure-ground, similarity, and continuity to deceive the predators’ brain. Conversely, successful identification of the cryptic animals requires the predators’ brain to actively apply these principles such as symmetry to extract the cryptic animals from their background. This competitive dynamic reveals the process of perception—the brain’s recognition and organization of sensations—and demonstrates how the competitive application of Gestalt principles leads to uncertainty in outcomes of perception, as well as the potential for resulting illusory interpretations. This empirical evidence supports the foundational definitions of perception and consciousness within the present framework (Martinez-Conde and Macknik, 2016; this study, Section 2.1).
Mirror test (mark test): Standard behavioral assay for visual self-recognition: an animal is marked on a body part not visible without a mirror; the test evaluates whether the animal uses its reflection to inspect the marked area, thereby providing evidence of self-recognition (Gallup, 1970).
Mirror reaction: The reaction of an animal’s brain to its own mirror image; a behavioral manifestation assessed in mirror self-recognition studies. In the present framework, differential mirror reactions across species serve as empirical indicators of distinct levels of consciousness, providing the observational basis for the distinction between BSC and MSC and grounding the Mirror-Induced Origination Hypothesis (this study).
Majority-failed-test (MFT) animals: The majority of animals that do not pass the standard mirror test (including monkeys, e.g., rhesus macaques). They possess BSC but are presumed to lack MSC (this study).
Handful-passed-test (HPT) animals: The minority of animals that pass the mirror test (e.g., chimpanzees, elephants, dolphins). They are posited to have reached at least Stage 6 of the MLC model (this study).
Extended mirror test with training: An experimental paradigm demonstrating that rhesus monkeys which initially fail the standard mirror test can acquire mirror-induced self-directed behaviors through long-term training. In this paradigm, the monkeys observe via a mirror the application of synchronous visual-somatosensory stimulation (e.g., a laser projecting irritating facial-tactile sensations) to their own body, combined with food rewards to reinforce visuo-somatosensory associations. After extensive training (12–38 days, 50–240 trials/day), some monkeys exhibit spontaneous, untrained mirror-induced self-directed behaviors (e.g., using the mirror to explore unseen body parts), indicating a transition from conditioned responses to behaviors resembling mirror self-recognition (Chang et al., 2015).
“Lakeside Image” thought experiment: A deductive thought experiment designed based on the Mirror-Induced Origination Hypothesis. By means of logical reasoning at the levels of individual behavior and brain cognition, it reconstructs the hypothetical process through which MSC might have emerged in an animal ancestor of humans. In the imagined scenario, an animal ancestor of humans encounters its own reflection in a lake and repeatedly interacts with the mirror image; from this premise, the experiment logically deduces a sequence of behavioral changes and corresponding conscious states, yielding eight successive stages of conscious transformation. This structured phenomenal sequence presents the MLC process, which provides the core framework for the subsequent theoretical account of MSC origination and its empirical validation (this study).
Rubber hand illusion (RHI) experiment: An experimental paradigm in which synchronous brushing is applied to a visible rubber hand and the participant’s hidden real hand for a period of time (typically 10 min), inducing an illusion that the rubber hand belongs to one’s own body. This paradigm provides key empirical evidence for the percept drift principle (Botvinick and Cohen, 1998).
Tubes task experiment: A typical metacognition paradigm assessing whether individuals seek information when uncertain about a hidden reward’s location. In this task, food or stickers are hidden in one of several opaque tubes; the key manipulation is whether subjects witness the baiting process. The critical measure is whether subjects spontaneously look into the tubes before making a choice. Great apes (chimpanzees, orangutans) and 2.5-year-old children look into the tubes significantly more often when they have not seen the baiting compared to when they have seen it, demonstrating that they can evaluate their own knowledge state and adaptively seek information to remedy ignorance—a foundational indicator of metacognitive ability (Call and Carpenter, 2001).
Multisensory integration mechanisms: Neurobiological substrate for percept drift, involving neurons and brain areas (e.g., premotor and parietal cortices) that integrate visual, tactile, and proprioceptive signals to generate a sense of body ownership and self-location (Blanke et al., 2015).

## References

[ref1] Andersen R. A. (1997). Multimodal integration for the representation of space in the posterior parietal cortex. Philos. Trans. R. Soc. Lond. Ser. B Biol. Sci. 352, 1421–1428. doi: 10.1098/rstb.1997.0128, 9368930 PMC1692052

[ref2] Anderson J. (1994). “The Monkey in the Mirror: A Strange Conspecific,” in Self-Awareness in Animals and Humans: Developmental Perspectives, eds. Parker S. Mitchell R. Boccia M. (Cambridge: Cambridge University Press), 315–329.

[ref3] Anderson J. R. Gallup G. G. (2015). Mirror self-recognition: a review and critique of attempts to promote and engineer self-recognition in primates. Primates J. Primatol. 56, 317–326. doi: 10.1007/s10329-015-0488-9, 26341947

[ref4] Aru J. Bachmann T. Singer W. Melloni L. (2012). Distilling the neural correlates of consciousness. Neurosci. Biobehav. Rev. 36, 737–746. doi: 10.1016/j.neubiorev.2011.12.003, 22192881

[ref5] Aspell J. E. Heydrich L. Marillier G. Lavanchy T. Herbelin B. Blanke O. (2013). Turning body and self inside out: visualized heartbeats alter bodily self-consciousness and tactile perception. Psychol. Sci. 24, 2445–2453. doi: 10.1177/0956797613498395, 24104506

[ref6] Baars B. J. (2009). “History of Consciousness Science,” in Encyclopedia of Consciousness, ed. William P. B. (Cambridge, MA: Academic Press), 329–338.

[ref7] Beran M. J. (2019). Animal metacognition: a decade of progress, problems, and the development of new prospects. Anim. Behav. Cogn. 6, 223–229. doi: 10.26451/abc.06.04.01.2019

[ref8] Blanke O. (2012). Multisensory brain mechanisms of bodily self-consciousness. Nat. Rev. Neurosci. 13, 556–571. doi: 10.1038/nrn3292, 22805909

[ref9] Blanke O. Slater M. Serino A. (2015). Behavioral, neural, and computational principles of bodily self-consciousness. Neuron 88, 145–166. doi: 10.1016/j.neuron.2015.09.029, 26447578

[ref10] Botvinick M. Cohen J. (1998). Rubber hands ‘feel’ touch that eyes see. Nature 391, 756–756. doi: 10.1038/35784, 9486643

[ref11] Buckner R. L. Bandettini P. A. O’Craven K. M. Savoy R. L. Petersen S. E. Raichle M. E. . (1996). Detection of cortical activation during averaged single trials of a cognitive task using functional magnetic resonance imaging. Proc. Natl. Acad. Sci. 93, 14878–14883. doi: 10.1073/pnas.93.25.14878, 8962149 PMC26230

[ref12] Call J. Carpenter M. (2001). Do apes and children know what they have seen? Anim. Cogn. 3, 207–220. doi: 10.1007/s100710100078

[ref13] Chalmers D. J. (1996). The Conscious mind. In search of a Fundamental Theory. Oxford: Oxford University Press.

[ref14] Chang L. Fang Q. Zhang S. Poo M. M. Gong N. (2015). Mirror-induced self-directed behaviors in rhesus monkeys after visual-somatosensory training. Curr. Biol. 25, 212–217. doi: 10.1016/j.cub.2014.11.016, 25578908

[ref15] Chella A. Frixione M. Gaglio S. (2008). A cognitive architecture for robot self-consciousness. Artif. Intell. Med. 44, 147–154. doi: 10.1016/j.artmed.2008.07.003, 18715770

[ref16] Chin J. M. Schooler J. W. (2009). “Meta-awareness,” in Encyclopedia of Consciousness, ed. Banks W. P., vol. 2 (Amsterdam: Elsevier), 33–41.

[ref17] Cohen Priva U. Austerweil J. L. (2015). Analyzing the history of cognition using topic models. Cognition 135, 4–9. doi: 10.1016/j.cognition.2014.11.006, 25497481

[ref18] Cohen J. D. Schooler J. W. (1997). Scientific Approaches to Consciousness. London: Psychology Press.

[ref19] De Croon G. C. H. E. Dupeyroux J. J. G. Fuller S. B. Marshall J. A. R. (2022). Insect-inspired AI for autonomous robots. Science. Robotics 7:eabl6334. doi: 10.1126/scirobotics.abl6334, 35704608

[ref20] Dehaene S. Changeux J. P. (2011). Experimental and theoretical approaches to conscious processing. Neuron 70, 200–227. doi: 10.1016/j.neuron.2011.03.018, 21521609

[ref21] Dehaene S. Lau H. Kouider S. (2017). What is consciousness, and could machines have it? Science 358, 486–492. doi: 10.1126/science.aan8871, 29074769

[ref22] Dehaene S. Naccache L. (2001). Towards a cognitive neuroscience of consciousness: basic evidence and a workspace framework. Cognition 79, 1–37. doi: 10.1016/S0010-0277(00)00123-2, 11164022

[ref23] Deroy O. Faivre N. Lunghi C. Spence C. Aller M. Noppeney U. (2016). The complex interplay between multisensory integration and perceptual awareness. Multisens. Res. 29, 585–606. doi: 10.1163/22134808-00002529, 27795942 PMC5082728

[ref24] Descartes R. (2016). *A discourse on method. Project Gutenberg e-text of a discourse on method by Rene Descartes*. Available online at: http://www.gutenberg.org/files/59/59-h/59-h.htm#part4.

[ref25] Dobzhansky T. (1967). The Biology of Ultimate Concern. New York: The New Press American Library.

[ref26] Dresp-Langley B. (2021). Consciousness beyond neural fields: expanding the possibilities of what has not yet happened. Front. Psychol. 12:762349. doi: 10.3389/fpsyg.2021.762349, 35082717 PMC8784399

[ref27] Eccles J. C. (1989). Evolution of the Brain: Creation of the Self. Abingdon: Routledge.

[ref28] Ehrsson H. H. (2007). The experimental induction of out-of-body experiences. Science 317:1048. doi: 10.1126/science.1142175, 17717177

[ref29] Engels F. (1995). The part played by labor in the transition from ape to man. Mon. Rev. 47:1. doi: 10.14452/MR-047-06-1995-10_1

[ref30] Eysenck M. W. Keane M. T. (2015). Cognitive Psychology: a Student’s Handbook. 7th Edn Abingdon: Psychology Press.

[ref31] Fetsch C. R. Deangelis G. C. Angelaki D. E. (2013). Bridging the gap between theories of sensory cue integration and the physiology of multisensory neurons. Nat. Rev. Neurosci. 14, 429–442. doi: 10.1038/nrn3503, 23686172 PMC3820118

[ref32] Flavell J. H. (1979). Metacognition and cognitive monitoring: a new area of cognitive–developmental inquiry. Am. Psychol. 34, 906–911. doi: 10.1037/0003-066X.34.10.906

[ref33] Frank M. J. Badre D. (2015). How cognitive theory guides neuroscience. Cognition 135, 14–20. doi: 10.1016/j.cognition.2014.11.009, 25496988 PMC4601572

[ref34] Gallagher I. (2000). Philosophical conceptions of the self: implications for cognitive science. Trends Cogn. Sci. 4, 14–21. doi: 10.1016/S1364-6613(99)01417-5, 10637618

[ref35] Gallagher S. (2007). “Phenomenological approaches to consciousness,” in The Blackwell Companion to Consciousness, ed. Velmanns M. (Hoboken, NJ: Blackwell Publishing), 686–696.

[ref36] Gallagher H. L. Happé F. Brunswick N. Fletcher P. C. Frith U. Frith C. D. (2000). Reading the mind in cartoons and stories: an fMRI study of “theory of mind” in verbal and nonverbal tasks. Neuropsychologia 38, 11–21. doi: 10.1016/S0028-3932(99)00053-6, 10617288

[ref37] Gallup G. G. (1970). Chimpanzees: self recognition. Science 167, 86–87. doi: 10.1126/science.167.3914.86, 4982211

[ref38] Goldstein E. B. Cacciamani L. (2022). *Sensation and Perception*. Cengage. Available online at: https://catalog.libraries.psu.edu/catalog/34874770.

[ref39] Goodale M. A. (2000). “Perception and action,” in Encyclopedia of Psychology, ed. Kazdin E., vol. 6 (Washington, DC: American Psychological Association), 86–89.

[ref40] Graziano M. S. (1999). Where is my arm? The relative role of vision and proprioception in the neuronal representation of limb position. Proc. Natl. Acad. Sci. U. S. A. 96, 10418–10421. doi: 10.1073/pnas.96.18.10418, 10468623 PMC17903

[ref41] Graziano M. S. Cooke D. F. Taylor C. S. (2000). Coding the location of the arm by sight. Science 290, 1782–1786. doi: 10.1126/science.290.5497.1782, 11099420

[ref42] Graziano M. S. Hu X. T. Gross C. G. (1997). Visuospatial properties of ventral premotor cortex. J. Neurophysiol. 77, 2268–2292. doi: 10.1152/jn.1997.77.5.2268, 9163357

[ref43] Green D. Swets J. (1966). Signal Detection Theory in Psychophysics. Hoboken, NJ: Wiley.

[ref44] Hare B. Call J. Agnetta B. Tomasello M. (2000). Chimpanzees know what conspecifics do and do not see. Anim. Behav. 59, 771–785. doi: 10.1006/anbe.1999.1377, 10792932

[ref45] Hoffmann M. Wang S. Outrata V. Alzueta E. Lanillos P. (2021). Robot in the mirror: toward an embodied computational model of mirror self-recognition. KI Künstliche Intell. 35, 37–51. doi: 10.1007/s13218-020-00701-7

[ref46] Hume D. (2000). A Treatise of Human Nature. Oxford: Oxford University Press.

[ref47] Iriki A. Tanaka M. Obayashi S. Iwamura Y. (2001). Self-images in the video monitor coded by monkey intraparietal neurons. Neurosci. Res. 40, 163–173. doi: 10.1016/S0168-0102(01)00225-5, 11377755

[ref48] Ishida H. Nakajima K. Inase M. Murata A. (2010). Shared mapping of own and others’ bodies in visuotactile bimodal area of monkey parietal cortex. J. Cogn. Neurosci. 22, 83–96. doi: 10.1162/jocn.2009.21185, 19199418

[ref49] James W. (1952). *The Principles of Psychology*. Encyclopedia Britannica.

[ref50] Kandel E. R. Squire L. R. (2000). Neuroscience: breaking down scientific barriers to the study of brain and mind. Science 290, 1113–1120. doi: 10.1126/science.290.5494.1113, 11185010

[ref51] Kline A. Forkert N. D. Felfeliyan B. Pittman D. Goodyear B. Ronsky J. (2021). fMRI-informed EEG for brain mapping of imagined lower limb movement: feasibility of a brain computer interface. J. Neurosci. Methods 363:109339. doi: 10.1016/j.jneumeth.2021.109339, 34454954

[ref52] Köhler W. (1940). *Dynamics in Psychology*. Liver.

[ref53] Krauss P. Maier A. (2020). Will we ever have conscious machines? Front. Comput. Neurosci. 14:556544. doi: 10.3389/fncom.2020.556544, 33414712 PMC7782472

[ref54] Kumar S. Hedges S. B. (1998). A molecular timescale for vertebrate evolution. Nature 392, 917–920. doi: 10.1038/31927 9582070

[ref55] Legrand D. (2006). The bodily self: the sensori-motor roots of pre-reflective self-consciousness. Phenomenol. Cogn. Sci. 5, 89–118. doi: 10.1007/s11097-005-9015-6

[ref56] Lopez C. Blanke O. (2011). The thalamocortical vestibular system in animals and humans. Brain Res. Rev. 67, 119–146. doi: 10.1016/j.brainresrev.2010.12.002, 21223979

[ref57] Lou H. C. Changeux J. P. Rosenstand A. (2017). Towards a cognitive neuroscience of self-awareness. Neurosci. Biobehav. Rev. 83, 765–773. doi: 10.1016/j.neubiorev.2016.04.004, 27079562

[ref58] Martinez-Conde S. Macknik S. L. (2016). Animal magicians. Scientific American. Mind 27, 18–19. doi: 10.1038/scientificamericanmind0316-18

[ref59] Noel J. P. Pfeiffer C. Blanke O. Serino A. (2015). Peripersonal space as the space of the bodily self. Cognition 144, 49–57. doi: 10.1016/j.cognition.2015.07.012, 26231086 PMC4837893

[ref60] Oizumi M. Albantakis L. Tononi G. Sporns O. (2014). From the phenomenology to the mechanisms of consciousness: integrated information theory 3.0. PLoS Comput. Biol. 10:e1003588. doi: 10.1371/journal.pcbi.1003588, 24811198 PMC4014402

[ref61] Palmer S. E. (2000). “Perceptual Organization,” in Encyclopedia of Psychology, ed. Kazdin E., vol. 6 (Washington, DC: American Psychological Association), 93–97.

[ref62] Pashler H. E. (1998). The Psychology of Attention. Cambridge: MIT Press.

[ref63] Patterson N. Richter D. J. Gnerre S. Lander E. S. Reich D. (2006). Genetic evidence for complex speciation of humans and chimpanzees. Nature 441, 1103–1108. doi: 10.1038/nature04789 16710306

[ref64] Petkova V. I. Ehrsson H. H. (2008). If I were you: perceptual illusion of body swapping. PLoS One 3:e3832. doi: 10.1371/journal.pone.0003832, 19050755 PMC2585011

[ref65] Plotnik J. M. de Waal F. B. M. Reiss D. (2006). Self-recognition in an Asian elephant. Proc. Natl. Acad. Sci. 103, 17053–17057. doi: 10.1073/pnas.0608062103, 17075063 PMC1636577

[ref66] Popper K. (1982). *The Open Universe: An Argument for Indeterminism*. Hutchinson.

[ref67] Qiu G. (2002). Mathematic Dictionary, vol. 1 Beijing: Science and Technology Press of China.

[ref68] Reiss D. Marino L. (2001). Mirror self-recognition in the bottlenose dolphin: a case of cognitive convergence. Proc. Natl. Acad. Sci. 98, 5937–5942. doi: 10.1073/pnas.101086398, 11331768 PMC33317

[ref69] Rhesus Macaque Genome Sequencing and Analysis Consortium (2007). Evolutionary and biomedical insights from the Rhesus macaque genome. Science 316, 222–234. doi: 10.1126/science.1139247, 17431167

[ref70] Rizzolatti G. Scandolara C. Matelli M. Gentilucci M. (1981). Afferent properties of periarcuate neurons in macaque monkeys. II. Visual responses. Behav. Brain Res. 2, 147–163. doi: 10.1016/0166-4328(81)90053-x, 7248055

[ref71] Rosenthal D. M. (2002). “Explaining Consciousness,” in Philosophy of Mind, ed. Chalmers D. J. (Oxford: Oxford University Press), 406–421.

[ref72] Seager W. (2009). “Philosophical accounts of self-awareness and introspection,” in Encyclopedia of Consciousness, ed. William P. B. (Cambridge, MA: Academic Press), 187–199.

[ref73] Sedekides C. Skowronski J. J. (1997). The symbolic self in evolutionary context. Personal. Soc. Psychol. Rev. 1, 80–102. doi: 10.1207/s15327957pspr0101_6, 15647130

[ref74] Signorelli C. M. Szczotka J. Prentner R. (2021). Explanatory profiles of models of consciousness – towards a systematic classification. Neurosci. Conscious. 2021, 1–13. doi: 10.1093/nc/niab021, 34457353 PMC8396118

[ref75] Slater M. Spanlang B. Sanchez-Vives M. V. Blanke O. (2010). First person experience of body transfer in virtual reality. PLoS One 5:e10564. doi: 10.1371/journal.pone.0010564, 20485681 PMC2868878

[ref76] Smith J. D. Beran M. J. Couchman J. J. Coutinho V. C. Boomer J. B. (2009). Animal metacognition: problems and prospects. Comp. Cogn. Behav. Rev. 4, 40–53. doi: 10.3819/ccbr.2009.40004

[ref77] Spence C. Driver J. (2004). Crossmodal Space and Crossmodal Attention. Oxford: Oxford University Press.

[ref78] Sternberg R. J. Sternberg K. (2012). Cognitive Psychology. 6th Edn Boston, MA: Cengage Learning.

[ref79] Suddendorf T. Butler D. L. (2013). The nature of visual self-recognition. Trends Cogn. Sci. 17, 121–127. doi: 10.1016/j.tics.2013.01.004, 23410584

[ref80] Tattersall I. (2004). What happened in the origin of human consciousness? Anatomical record. Part B New Anatomist 276, 19–26. doi: 10.1002/ar.b.10041, 14750191

[ref81] Tsakiris M. (2008). Looking for myself: current multisensory input alters self-face recognition. PLoS One 3:e4040. doi: 10.1371/journal.pone.0004040, 19107208 PMC2603324

[ref82] Tsakiris M. Haggard P. (2005). The rubber hand illusion revisited: Visuotactile integration and self-attribution. J. Exp. Psychol. Hum. Percept. Perform. 31, 80–91. doi: 10.1037/0096-1523.31.1.80, 15709864

[ref83] Tsakiris M. Hesse M. D. Boy C. Haggard P. Fink G. R. (2007). Neural signatures of body ownership: a sensory network for bodily self-consciousness. Cereb. Cortex 17, 2235–2244. doi: 10.1093/cercor/bhl131, 17138596

[ref84] Watson J. B. (1914). Behavior: An Introduction to Comparative Psychology. New York: Henry Holt and Com.

[ref85] Wilson D. S. Sober E. (1994). Reintroducing group selection to the human behavioral sciences. Behav. Brain Sci. 17, 585–608. doi: 10.1017/S0140525X00036104

[ref86] Zimbardo P. G. Johnson R. L. McCann V. (2014). Psychology: Core Concepts. 7th Edn Beijing: Pearson Education Asia Ltd., and Tsinghua University Press.

